# Un/met: a mixed-methods study on primary healthcare needs of the poorest population in Khyber Pakhtunkhwa province, Pakistan

**DOI:** 10.1186/s12939-024-02274-5

**Published:** 2024-09-23

**Authors:** Maira Shaukat, Alina Imping, Lisa Rogge, Fatima Khalid, Safat Ullah, Fayaz Ahmad, Zeeshan Kibria, Andreas Landmann, Zohaib Khan, Manuela De Allegri

**Affiliations:** 1 Heidelberg Institute of Global Health, Medical Faculty and University Hospital, Heidelberg University, Heidelberg, Germany; 2 Friedrich-Alexander University of Erlangen-Nürnberg, Erlangen, Germany; 3 Institute of Public Health & Social Sciences, Khyber Medical University, Peshawar, Pakistan; 4 Office of Research, Innovation, and Commercialization (ORIC), Khyber Medical University, Peshawar, Pakistan

**Keywords:** Universal health coverage, Universal health insurance, Primary health care, Access to primary care, Health equity, Needs assessment pakistan

## Abstract

**Background:**

Access of all people to the healthcare they need, without financial hardship is the goal of Universal Health Coverage (UHC). As UHC initiatives expand, assessing the needs of vulnerable populations can reveal gaps in the system which may be covered by relevant policies. In this study we (i) identify the met and unmet primary healthcare needs of the poorest population of Khyber Pakhtunkhwa province (KP), Pakistan, and (ii) explore why the gaps exist.

**Methods:**

We used Leveque’s Framework of Patient-centred Access to Healthcare to examine unmet primary healthcare (PHC) needs and their underlying causes for the poorest population in four districts of Khyber Pakhtunkhwa province, Pakistan. Using a triangulation mixed methods design, we analysed data from a quantitative household survey of744 households, 17 focus group discussions with household members and, 11 interviews with healthcare providers.

**Results:**

Our results show that indicate that despite service utilization, PHC needs were not met, primarily due to prohibitively high costs at each stage of access. Furthermore, gaps in outreach and information (approachability), and varying availability of medicines and diagnostics at facilities (appropriateness) the supply side as well as difficulties in navigating the system (inability to perceive) and adhering to prescriptions (inability to engage) on the demand side, also led to unmet PHC needs. Going beyond utilization, our findings highlight that engagement with care is an important determinant of met needs for vulnerable populations.

**Conclusion:**

Social health protection policies can contribute to advancing UHC for primary care. However, in our setting, enhancing communication and outreach, addressing gender and age disparities, and improving quality of care and health infrastructure are necessary to fully meet the needs of the poorest populations.

**Supplementary Information:**

The online version contains supplementary material available at 10.1186/s12939-024-02274-5.

## Highlights

• The primary health needs of the poorest population of Khyber Pakhtunkhwa remain largely unmet or are met with difficulties, across the care-seeking pathway.

• Major contributors to unmet primary healthcare needs in the study area are: low outreach, crowded facilities and, varying availability of medicines and diagnostics at facilities on the supply side, and difficulties in navigating care and inability to afford prescribed care on the demand side.

• Social Health Protection is a crucial tool for advancing Universal Health Coverage and improving primary healthcare, however these needs cannot be met without quality service provision and outreach from facilities.

• Levesque’s framework of access to care (2013) can be effectively employed to identify broad-based unmet needs and assess gaps in primary healthcare systems.

**Supplementary Information:**

The online version contains supplementary material available at 10.1186/s12939-024-02274-5.

## Background

Poverty increases health vulnerability two-fold: on one hand, poorest population segments have worse health outcomes and greater need; on the other, they are more likely to face access barriers [1]. As countries across the world strive to achieve the goal of Universal Health Coverage (UHC) is defined as access of all people to the healthcare they need, without incurring financial hardship [2]. Based on sustainable development goal 3.8 [3] countries across the world are striving to achieve UHC. Identifying unmet health needs of the poorest is key to ensure equitable inclusion of this vulnerable group in UHC initiatives.

Social health protection (SHP) programs are implemented as to facilitate UHC through financial risk protection [4–7]. In South Asia, countries have adopted SHP policies, with a common starting point often being secondary and tertiary (inpatient) care coverage [7, 8]. As full effects of these programs are calibrated, extending them to include primary care could offer higher financial protection as evidence shows that outpatient care constitutes the largest portion of healthcare expenditures in this region [9, 10]. The importance of primary healthcare (PHC) systems to deliver basic healthcare for all was highlighted in the Alma Ata declaration in 1978, and described as socially acceptable, universally approachable, and scientifically sound care [11]. Since then, progress towards strengthening PHC systems has been slow [12]. In the recent years, including in the wake of the COVID-19 pandemic, the unexploited potential of PHC systems to meet the needs of vulnerable populations has come to the forefront again offering opportunities for primary care programs to prioritize it [13]. Identifying the needs of vulnerable groups, can support in designing equitable PHC initiatives, and understanding the nature of unmet needs can elaborate necessary mechanisms to achieve this.

Unmet needs in primary care have been examined frequently in higher-income settings [14–18] but evidence from low-and-middle income countries (LMICs) remains sparse; most published peer-reviewed literature is relatively recent [19–25]. Specifically examining PHC needs, one study from Kenya identifies moderate unmet PHC needs with women, the elderly, and less wealthy groups more likely to forgo care [23]. A study from India elicited high unmet needs for non-communicable disease (NCD) diagnosis [26], and recommended qualitative approaches in future studies to understand appropriateness of care in relation to unmet needs [26]. Evidence from Pakistan describing PHC unmet needs is also sparse. One study highlighted gender related difference in access to PHC in Balochistan province [27]. A systematic review on women’s PHC access barriers, identified cultural and health service related barriers [28]. While supply and demand side factors are mentioned in these studies, the link between them resulting in unmet needs has not been established. The influence of poverty is also not the focus of these studies.

In this study we (i) identify the met and unmet PHC need of the poorest population of Khyber Pakhtunkhwa (KP) province, Pakistan, and (ii) explore why the gaps exist. Through this, we offer recommendations for UHC policymakers to design equitable PHC initiatives. The context in KP of an emerging SHP system partially covering secondary and tertiary services, but not PHC, is furthermore representative for the challenges many LMICs face currently [9]. Given that it has a weak, but existent PHC system – a well-known phenomenon around the world [29] - KP can offer insights for other similar settings. We applied a mixed-methods approach [30], with demand and supply side data to provide perspectives *of* and *on* the end-users of the healthcare system, respectively, to consider primary care health needs, and their complexity using Levesque’s Framework of Access to Patient Centred Health care [31].

## Methods

### Study setting

Pakistan has made significant progress towards UHC in the past decade [32] although indications of unmet needs have been made by previous studies [33]. The public health infrastructure consists of three tiers of facilities offering highly subsidized rates at the point of care [34]. A largely unregulated private sector thrives due to low capacities of public facilities [34]. Average out of pocket expenditures were 53.2% in 2019-20 [35]. An Essential Package of Health Services was presented in 2021 as high priority and high cost-effectiveness interventions to be covered under UHC programs [36].

A donor supported SHP program was launched in 2015 in KP followed by other regions of Pakistan [37]. Offering financial coverage for inpatient procedures, these programs started with covering the poorest income quintile of the population [37]. The government of KP, wants to extend its SHP program coverage to include outpatient department (OPD) services for the poorest population, with a PHC strengthening focus. The scheme – called Social Health Protection Initiative phase-II (SHPI-II) – is planned for a 2024 pilot, and is supported with financial contributions from the German government.

**Fig. 1:**
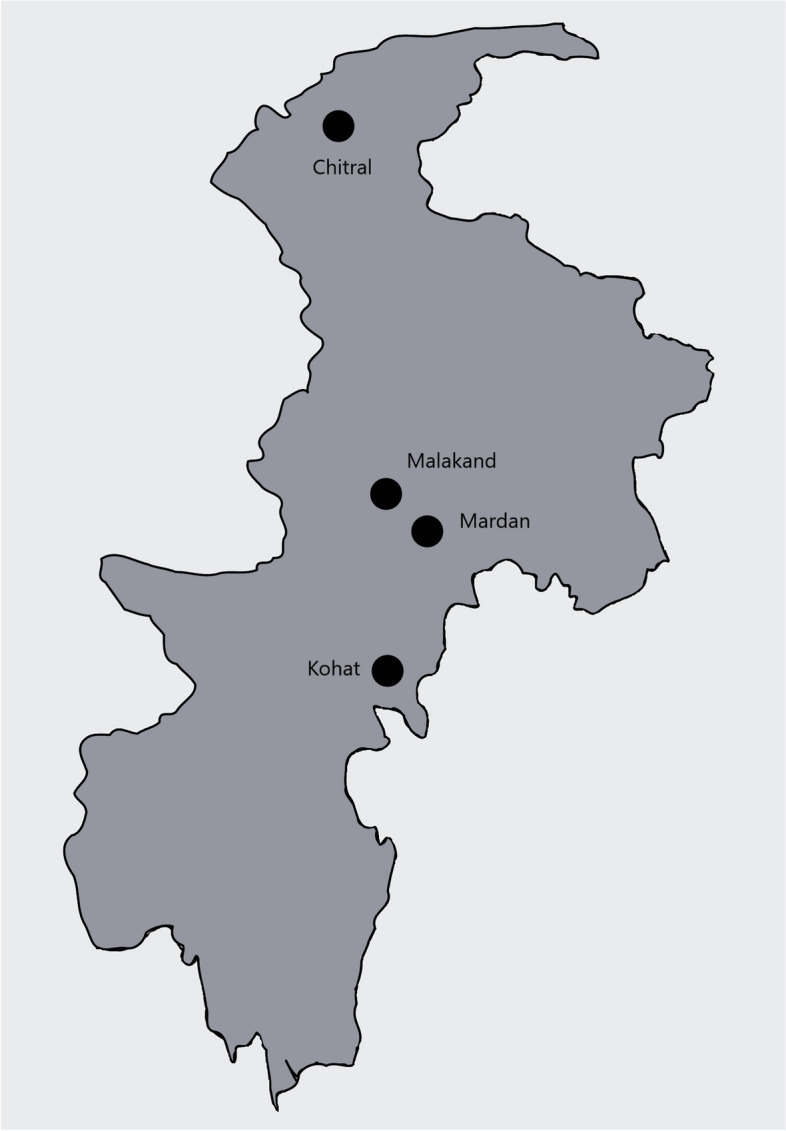
Map of Khyber Pakhtunkhwa province with location of selected districts

We conducted this study as part of the INSPIRE Pakistan research consortium [38] to support the implementers in the process of developing and implementing the SHPI-II. We therefore collected data in four districts of KP, where SHPI-II will be launched (Fig. 1). These districts are diverse in terms of geography and health infrastructure, helping us profile a variety of experiences within the poorest population of KP. The target population of our study were the potential beneficiaries of the SHPI-II program, who have been set to be the poorest (21%) households in the four districts. Eligibility information on these households was drawn from the Proxy Means Test scores lists prepared for the Benazir Income Support Programme (BISP) [39] in 2010, which were considered to be used for the SHPI-II program at the time of the study. Being asset-based the BISP scores are a more accurate measure of socio-economic deprivation than income level, useful for identifying the most vulnerable population.

### Conceptual framework

#### Unmet needs

Unmet needs are *sociomedical*: they present both at the social level as well as in the state of an individual’s physical health [40]. They are described as, ‘the differences, if any, between those services judged necessary to deal appropriately with defined health problems and those services actually being received’ [40]. The spectrum of needs, thus, is influenced by factors *of* and *beyond* medical care. Recent scholarship considers subjective approaches (described by individuals themselves) to understanding unmet needs superior as they can draw on perceived needs that were not brought to care or ‘forgone’ [14, 41, 42]. This also acknowledges ‘people-centeredness’, which has consistently been encouraged in PHC systems [29, 43]. People-centredness can guide research and practice communities towards creating structures and processes which are based on peoples’ needs, angled towards fulfilling them [29, 43]. We therefore centered our study on subjective unmet needs in line with our objectives.

#### Levesque’s framework of access to care

**Fig. 2:**
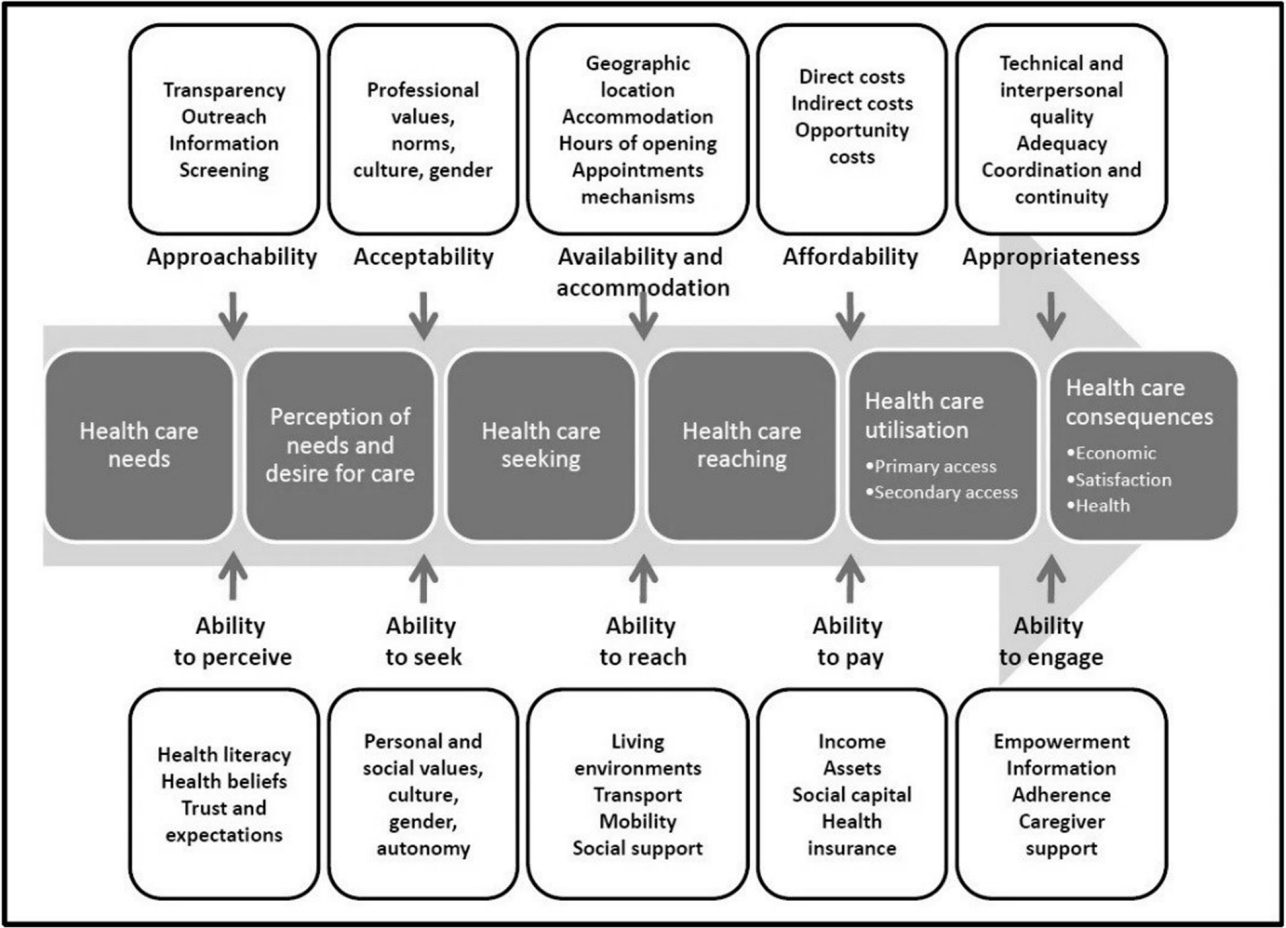
Levesque et al.’s framework of access to care (Levesque et al. 2013) [31]

We conceptualized needs as antecedent to access using Levesque’s framework of patient-centred access to care [31] (Fig. 2). The framework presents access to healthcare on a pathway; to move from one stage to another, the ability of an individual and the capacity of the system must align, categorizing clearly where the met and unmet needs can lie.

We used this framework as it conceptualizes health needs on a continuum over the course of care-seeking, compared to other frameworks of access [44], which refer to needs at a fixed moment during care seeking. Secondly, the role of the supply and demand side in meeting needs is crucial to this framework, and our research.

Not all stages of the framework were empirically examinable in the quantitative model as comprehensively as we conceptualized them. We operationalized two main concepts in the following ways: utilization was defined as care seeking at a public or private health facility; forgone care was any time a person considered seeking care but did not go to a formal facility. We categorized health facilities as higher level including secondary and tertiary care, and primary care facilities.

It is important to acknowledge that in our setting people may seek primary care at ‘outpatient departments’ (OPD) located at different levels, as well as in the private sector. Basic healthcare in Pakistan is not always accessed at the primary level, and people may directly approach higher levels [45, 46]. We hence referred to primary care as OPD care during data collection to align our language with the emic understanding of our respondents.

### Study design

We chose a mixed-methods approach to strengthen the validity and decrease the limitations of inherent in each method [47]: qualitative data provide in-depth explanatory and exploratory insights into the framework dimensions, while quantitative data offer an overall picture of met and unmet needs. We collected data for the two components separately and interpreted results jointly, i.e., methodological triangulation [48].

### Data Collection

We conducted two focus group discussions (FGD) each, with male and female respondents in all four districts, using a purposive sampling strategy and sought saturation within and across districts -one additional FGD was conducted with men in Chitral, for a total of 17 FGDs. All FGDs included 5–8 participants, representing a potential beneficiary household, preferably aged over 35 years to report for the entire household. We used an FGD guide (see Additional File 1) based on the framework. We conducted in-depth interviews (IDI) with one physician each at a public primary, public higher level, and private facility, in all districts. After refusal from one public higher-level facility, we completed 11 in-depth interviews. We interviewed physicians among healthcare providers owing to their higher access to information about administration and regulations. A semi-structured interview guide based on the framework was used (see Additional File 1).

We drew respondents for the quantitative survey via random sampling from the list of potential beneficiaries in all four districts restricting to accessible union councils (UC) considering safety, weather, and distance to a potentially covered health facility. Among these, the sample was randomly drawn in three stages: four UCs per district, up to four villages per UC, then households within these villages (see Additional File 2).

We interviewed one main respondent per household in an in-person interview (January-March 2022), who answered questions for him/herself and their household members. In addition, we collected detailed information on the health needs of children via a follow-up phone survey (May 2022), for which we reached almost 90% of respondents from the in-person interviews. The final sample comprised 744 households with a total of 4,017 family members^1^. The survey (see Additional file 4) was administered via a tablet-based questionnaire.

Data were collected between December 2021, and May 2022. All tools were translated into Urdu and Pashto and piloted before formal data collection. Data were collected by trained graduate and undergraduate level research assistants, well-versed in local languages and dialects. We obtained written consent of all participants, for qualitative data also to record audio until *verbatim* transcription. There was no overlap between the qualitative and quantitative samples, not to exhaust the respondents.

### Data Analysis

Transcripts of FGD’s and IDI’s were translated into English. Three researchers (MS, FK, and SU) systematically coded them in Nvivo 13 using a deductive codebook, reflecting the Levesque’s framework, with a few codes added inductively. We conducted an interrater reliability exercise on three test transcripts until high agreement was reached. We concentrated all information from the codes into Microsoft Excel and after three stages of concentrating data, presented the main findings, with examples and descriptions under each of the 10 dimensions of the framework.

The main measure of primary care need in the quantitative survey was self-reported health care utilization. We recorded the number of OPD visits and neglected OPD visits within a one-month recall period differentiated by facility types (higher/lower level, public/private) and reasons for seeking care for each core family member. We separately recorded pharmacy visits and asked for other health problems where seeking care was not considered. For survey time reasons, we only recorded out-of-pocket expenditures for the most recent OPD case per person, which we consider to be representative of all health needs of the respective person.

Survey data were analysed descriptively along the framework dimensions to describe the process of care step-by-step. In the first dimension, we added a logit regression, in which we used need for care as an outcome and age, gender, health status, district, distance to next health facility and a wealth index as explanatory factors. We chose the respective factors based on common factors to explain health need from the literature and analytical hints derived from the qualitative analysis. All reported figures accounted for sampling weights to factor differing sampling probabilities of target households across districts, UCs and villages. We conducted all quantitative analyses in Stata 16. For estimation of the logit regression we used the logit-command and clustered standard errors at the household level. For other descriptive analyses, we derived frequencies and estimated weighted means, for which we reported point estimates and 95% confidence intervals where appropriate.

After initial analysis, qualitative and quantitative findings were integrated using a narrative *weaving* approach [49].

## Results

A total of 129 people participated in focus group discussions of whom 67 were male and 62 were female. Eleven healthcare practitioners participated, two of whom were female. Age data for practitioners was not collected due its irrelevance with the study objectives. In the household survey 744 households were represented with an average of 4.05 adult members and 1.62 under 15. The average monthly out-of-pocket health expenditures were PKR 38,106. The characteristics of respondents of both study components are shown in Tables 1 and 2.

**Table 1:** Qualitative sample characteristics

Characteristics	Focus Group Discussion (*n* = 129)	In-Depth Interview (*n* = 11)
*Age*		
< 35	5	n/a
35–49	49	
50–65	62	
> 65	13	
*Sex*		
Female	62	2
Male	67	9

**Table 2:** Quantitative sample characteristics

		N	Mean	Standard Deviation	Minimum	Maximum
*Household characteristics*						
Household members		744	6.86	2.67	1	21
Core family adults		744	4.05	1.59	1	9
Core family children (< 15 years)		744	1.62	1.63	0	8
Wealth (PMT^a^ score)		744	11.58	3.19	0.02	16.14
Monthly expenditure (PKR)		734	38,106	18,423	1,500	120,400
Years enrolled in IPD^b^ insurance		710	5.71	0.41	4.56	5.92
*Respondent characteristics*						
Age (years)		743	47.23	15.04	16	96
Female		744	0.33		0	1
Any formal education		743	0.34		0	1
Married		744	0.81		0	1
Household financial decision-maker		744	0.70		0	1
Working		744	0.44		0	1

^a^Proxy Means Test

^b^In Patient Department

### Utilized and forgone OPD care

From the household survey, we saw the following patterns: The majority of families (82.38%) and 41.28% of individuals reported at least one OPD care visit within the past month. On average, we recorded 0.85 OPD visits per person per month to formal care facilities. Almost half of the families (48.22%) reported at least one case where they considered seeking OPD care but did not go within the past month. On the individual level, this corresponded to 15.45% of the 4,017 family members or a per-person average of 0.37 forgone OPD visits within the past month.

**Fig. 3:**
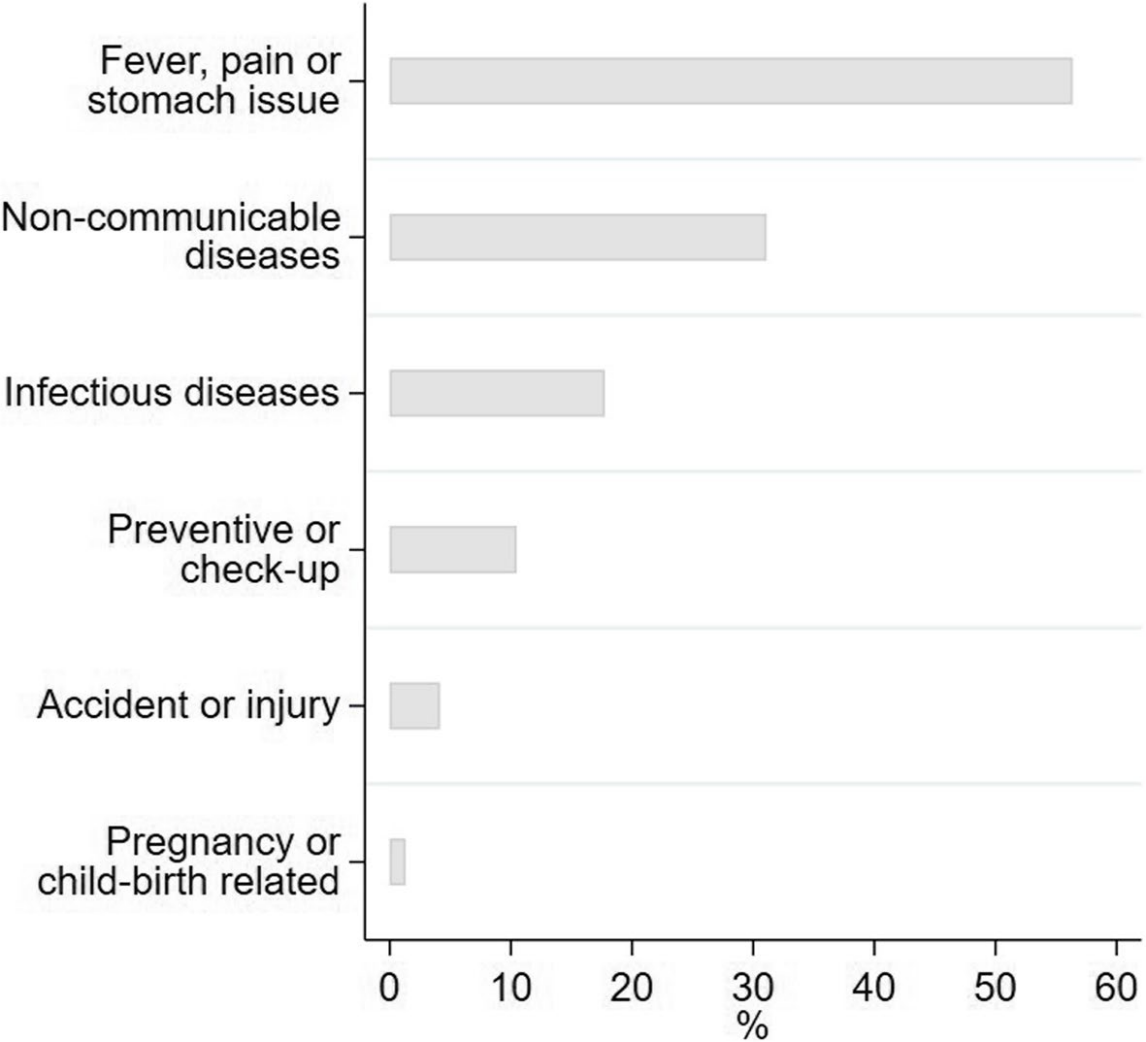
Reasons for seeking OPD care. Bars represent the weighted percentage of persons who reported the respective reason for seeking OPD care among those with at least one OPD visit within the past month

**Fig. 4:**
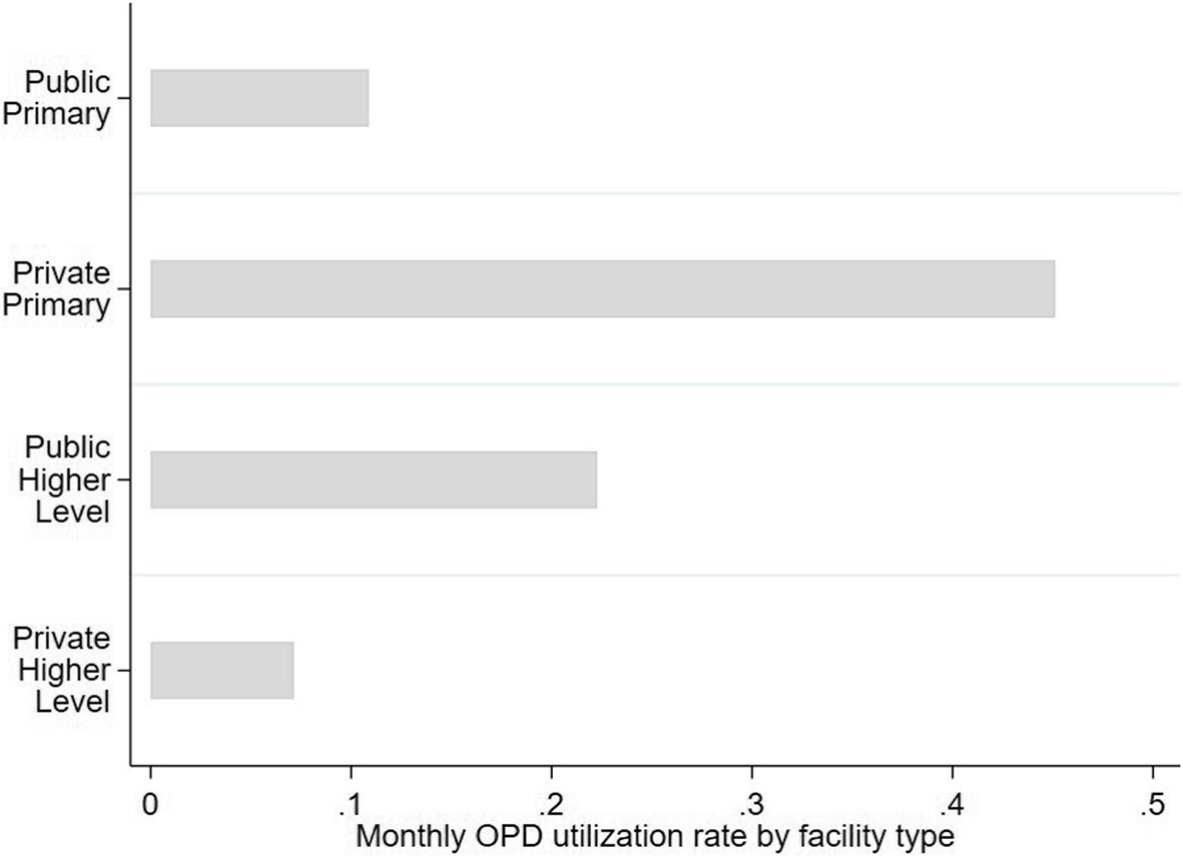
Monthly OPD utilization rate by facility type. Bars represent the average monthly OPD utilization rate (weighted mean) by facility type based on all core family members

Survey data revealed that the majority of OPD visits (58%) were due to acute illnesses. However, substantial preventive visits (12%) and a few accidents or injuries (5%) were also reported. Only a small fraction of respondents reported pregnancy or childbirth related OPD visits (2%) (Fig. 3). Private primary-level facilities were most frequented (45%), followed by public higher-level facilities (22%) (Fig. 4).

Participants in the qualitative component mentioned that some health needs were neglected while for others care was simply sought later i.e., delayed care seeking:

*‘We tolerate pain and resist our medical condition until we can*,* but if condition gets worst then we have to take loans and pay for our treatment. For relieving pain*,* we take painkillers.’* (Female, Chitral).

Self-care during this period did or did not not alleviate the need, and physicians attributed delayed care-seeking to their poorest patients especially, which sometimes resulted in complications for example, for pregnant women.

### Understanding unmet needs

#### Perception of needs and desire for care

At the first stage, health needs require perception, followed by decisions for pursuing care which are based on knowledge of healthcare and desire for care, respectively.

FGD respondents had a basic awareness of health conditions, diagnostics, and medication i.e. they could name but not explain the underlying mechanisms and connections between them. Common information sources were word-of-mouth, and previous experiences. The practitioners interviewed described that while their patients from low economic segments displayed a good vocabulary regarding diseases and healthcare, they did not have accurate knowledge about the extent of their illness and the need for immediate care; poorer patients visited with exacerbated symptoms, compared with others. The physicians however also acknowledged that the information and outreach functions of public facilities were not being exploited well e.g., one interviewee remarked that facility waiting rooms could use multimedia to deliver messages about care and prevention, but this was not being practised.

*‘One more thing that is even more important is that we should have health educators*,* female and male in every waiting area. They would guide people about the disease and tell them how to manage and what changes to bring in their lifestyle for the management of the disease.’* (Physician, Public secondary facility, Kohat).

Respondents explained that they took rest at the first recognition of illness, tried home remedies such as drinking tea, and if symptoms subsided, no formal care was sought. In our household survey, we asked for health events where seeking formal care was not considered: around 19.5% of family members reported such a health need, which they managed through self-medication including purchasing medicine without a prescription, home remedies, spiritual healing, and prayer, or doing nothing. Alternative care was reported by FGD respondents and in survey data but availing it was not a substitute for, but in addition to biomedical care, and predominantly for chronic conditions.

FGD respondents remarked that lack of accurate information negatively affected their ability to navigate the health system. To reach the first point of care, the choice of provider was made by the patient and their family, as referrals are not required; leading to patients being turned over from facility to facility. Decisions about where to seek care were based on previous experiences, the reputation of the physician, and recommendations from friends or family.

*‘*[My daughter] *sometimes went to one doctor and sometimes to another. Some doctors said that she had pain in the intestine*,* some doctors said there is swelling in her uterus… After that some doctors said she had only 3 days left to live. Different doctors have different comments.’* (Female, Mardan).

The desire for seeking facility-based care was also affected by trust in the system as a whole, although in the quantitative survey, trust in physicians was reported to be high (see Additional File 3, Table 1). There was an understanding among the FGD respondents that the health system favoured the rich and the well-connected, and that poor people could not expect any support. People mentioned feeling coerced to use low quality health services compared to others.

‘*In public hospitals they have such big egos that they won’t even look at a poor patient. That’s why I don’t go to the hospital.*’ (Female, Kohat).

When explaining reported outpatient need in the quantitative survey data in a multivariable regression we saw that (see Additional File 3, Table 2): households having more older or female members, characteristics that are expected to be associated with higher health need, had significantly higher odds of reporting a need (age: OR 2.83 95% CI 1.95–4.09, female gender: OR 1.316 95% CI 1.0-1.66). On the other hand, higher wealth was also associated with higher odds of reporting a need (OR 1.36 95% CI 1.18–1.157), which is in line with the hypothesis that the poor might exhibit a lower desire to seek care.

#### Healthcare seeking

After the desire for seeking care is established, the Leveseque’s Framework of access to care considers social, cultural, and individual factors which may influence access.

**Fig. 5:**
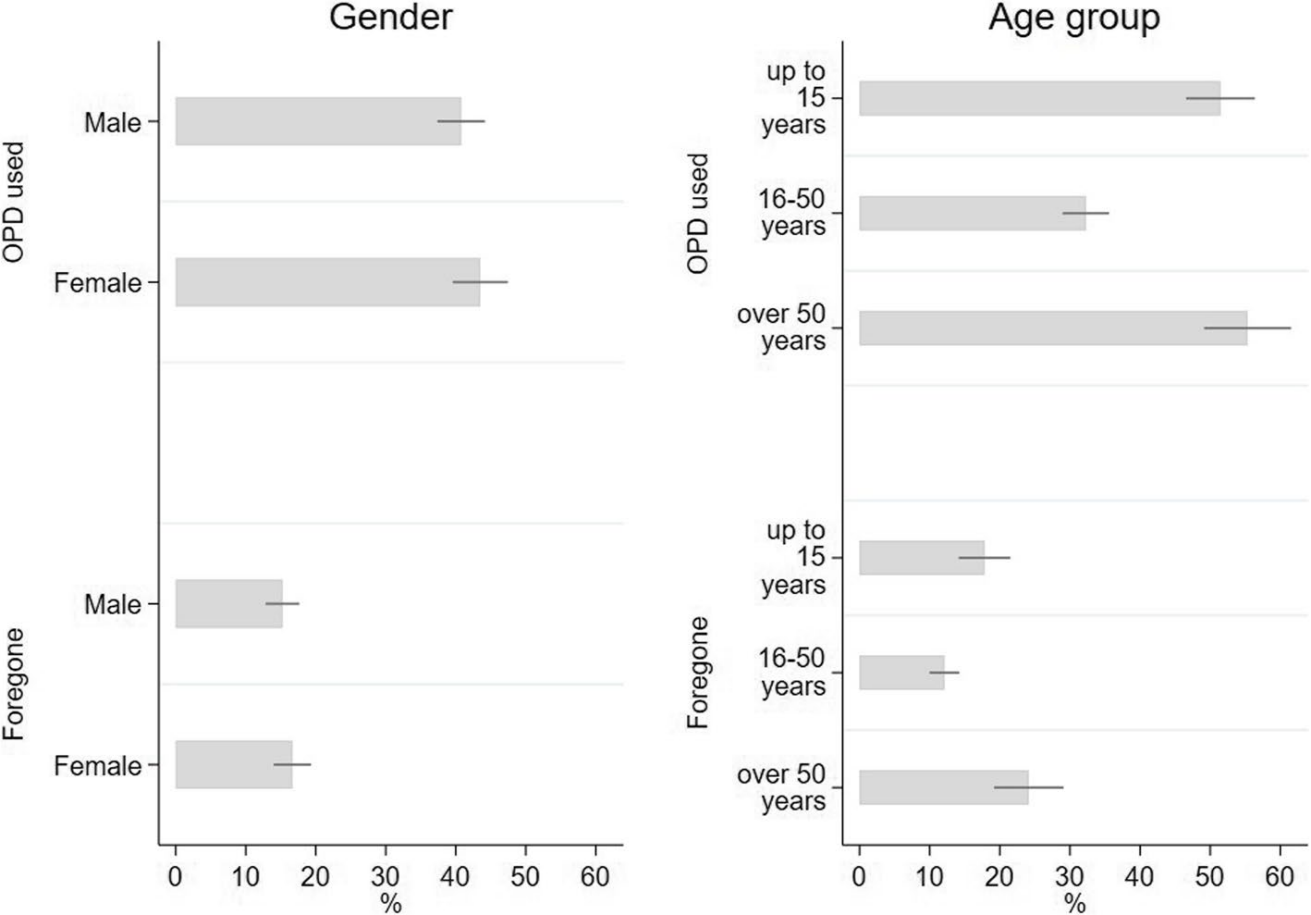
Utilized and forgone care by age and gender. Bars represent the average monthly OPD utilization rate based on all core family members by gender (upper left panel) and age groups (upper right panel) and average monthly foregone OPD visits based on all core family members by gender (lower left panel) and age groups (lower right panel); with 95% confidence interval around weighted mean

 Examining utilization by gender, the quantitative data showed similar levels of healthcare utilization for female (44%) and male family member (40%) and the same applied to foregone care (males 15%, females 17%) (Fig. 5). Discussing barriers to care seeking, female FGD respondents mentioned that transport was more expensive for them, as they preferred not to use public transport. The quantitative survey data confirmed that on average, transportation costs for women are higher, yet not statistically significantly different from those of men (see Additional File 3, Table 3). In addition, there were difficulties in leaving household chores and children behind, even though support was available from neighbours or relatives, and they mentioned lack of autonomy to reach all aspects of healthcare independently such as buying medication, or visiting facility without male family members.

*‘If a male* [relative] *is not with us then we have to ask some other person or request them to please bring medicines for us.’* (Female, Kohat).

Men reported delaying or forgoing care due to the opportunity costs of skipping work, as a significant portion of the respondents was employed in the informal sector.

 In survey data on health decision-making, while 71% male adult family members reported to be decision-makers, only 40% of females did (see Additional File 3, Table 4). FGD respondents explained that health decision-making was actually a collective process within families, but the final decision lay mostly with the older male household head.

 The position of an individual in the family also determined if care would be received; respondents mentioned doing everything in their capacity to ensure care of children and the elderly, even if it cost more. For example, the elderly had higher transport expenditures on average (PKR 309 vs. PKR183) (see Additional File 3, Table 5). Women reported forgoing care to save resources for their children. We did not see large differences in utilization across age groups in survey data, but children (up to 15 years) and older people (above 50 years) reported to use and to forego OPD care more (Fig. 5).

In terms of supply side consideration of social and individual factors, physicians mentioned that many primary care facilities did not have female health service providers, particularly laboratory technicians, however, male staff was not observed as prohibitive for women to seek care, except for gynaecological issues. While gender segregation norms were said to be respected, some facilities did not have enough resources to ensure this.

*‘If a female requires a detailed examination*,* I shall take her to LHV’s* [Lady Health Visitor] *room rather than do the inspection here… but there are occasions when I am obliged to use another bed since the female bed is filled.’* (Public primary facility, Chitral).

No special age-related services were mentioned.

#### Healthcare reaching

Physical access to care is multifaceted and an important stage in the care seeking pathway to examine systemic gaps.

**Fig. 6:**
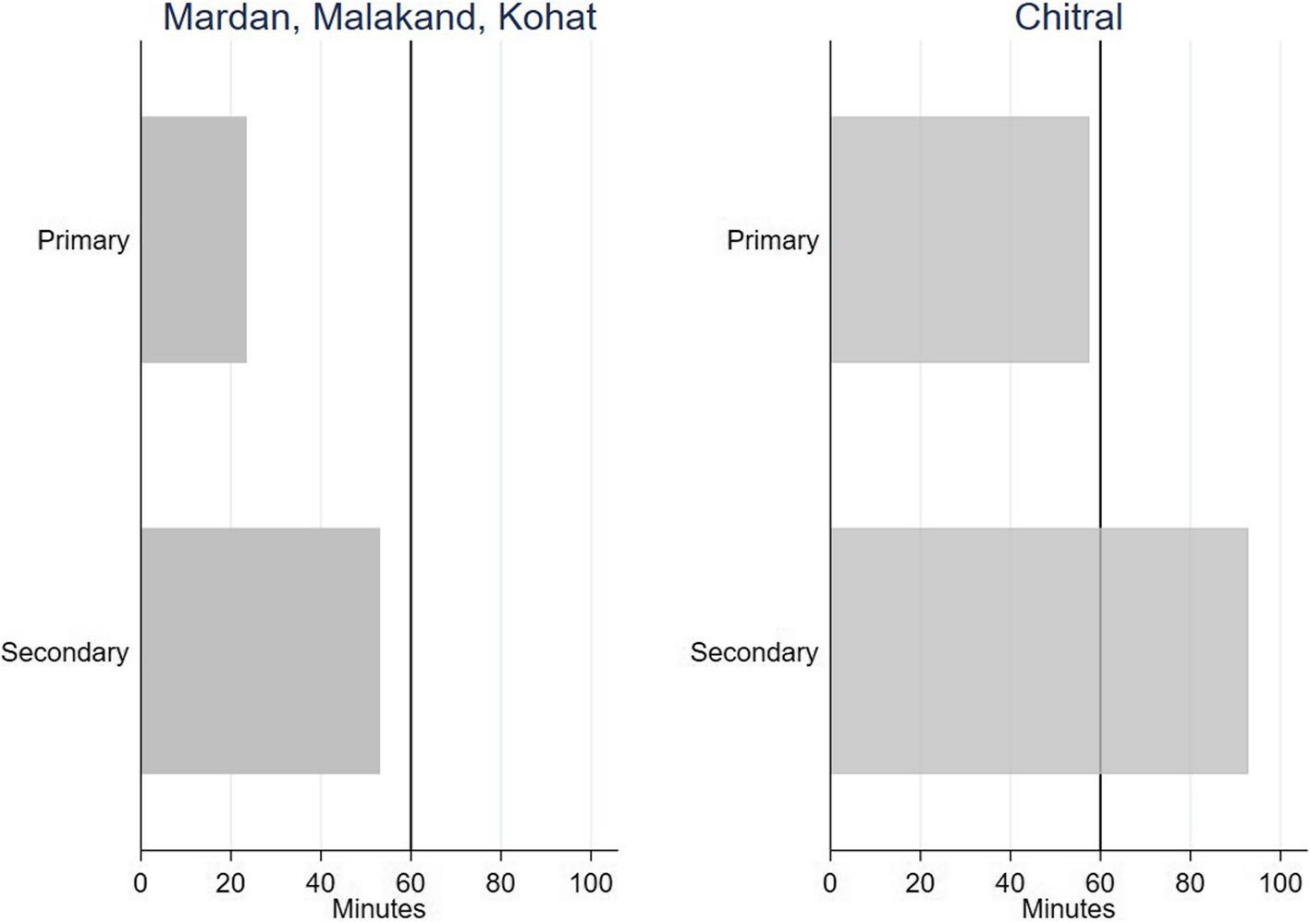
Self-reported travel time to reach nearest facility. Bars represent the average self-reported travel times (weighted mean) to the nearest health facility respondents reported in minutes by facility types for district Chitral (right panel) and the other districts (left panel)

 Our household survey measured the distance of the household to any type of health facility, in minutes (by usual mode of transport). It revealed that the study population had physical access in terms of distance as self-reported travel time to reach care was less than one hour, and substantially lower for primary facilities for most respondents (Fig. 6). However, people associated public facilities, particularly higher-level facilities, with long queues, extensive waiting, and some people recalled missing the consultation due to overcrowding. Waiting times hindered particularly those with informal jobs, from visiting a facility. Specialist OPD care and diagnostics were only available in public facilities on certain days of the week, coercing people to choose private care for urgent treatment or for seeking care in the evening hours. People recounted travelling frequently to the provincial capital and other cities to get appropriate care or diagnostics, and chronically ill respondents had higher costs of transport (see Additional File 3, Table 6). Acknowledging this, physicians shared that the patients begin to queue before the opening hours even started yet owing to high patient load in combination with time spent on administrative tasks, there was nothing they could do to improve the situation. Emergency services were available round the clock in higher level facilities, but primary level facilities only operated during the day.

In Chitral, FDG respondents and physicians alike remarked on the remoteness of the area making care seeking highly subject to the weather and road conditions.

‘*Right now*,* there is no snow in winters but otherwise all the roads get blocked.*’ (Public secondary facility, Chitral).

Quantitative data on utilization by level revealed that in the previous month, people visited private primary care facilities most often (45%), followed by public higher-level facilities (22%), public primary (11%) and lastly, private higher-level facilities (7%) (Fig. 4). FGD respondents explained preferring public facilities as they were the cheapest overall, including specialized care and subsidized diagnostics, yet, private primary care facilities were most frequented due to their proximity, and familiarity with the provider. Respondents acknowledged that private primary facilities may be run by unqualified practitioners i.e. ‘village doctors’. In one FGD respondents proposed the idea of decentralized public primary facilities to save costs of travelling, Interviewed physicians acknowledged the variable availability of services in public primary facilities.

Another aspect of managing care access in our setting were community members who provided substantial support by offering or, arranging for transport, and accompanying the patient to the facility.

*‘Our community’s tradition dictates that in an emergency*,* everyone with a car will assist in transporting the patient. And the volunteers are so numerous that if the patient is unable to pay*,* they will spend from their own pockets.’* (Male, Kohat).

As patient attendants community and family members supported patient needs and, in some cases, even made the follow-up facility visits. Many respondents mentioned using social connections to skip queues and get medicines from limited stocks, and preferred to visit facilities where they knew someone.

#### Healthcare utilization

Costs of seeking care are the fundamental concern of SHP policies, and the Levesque’s framework of access to care not only considers direct, but also indirect costs of seeking care.

**Fig. 7:**
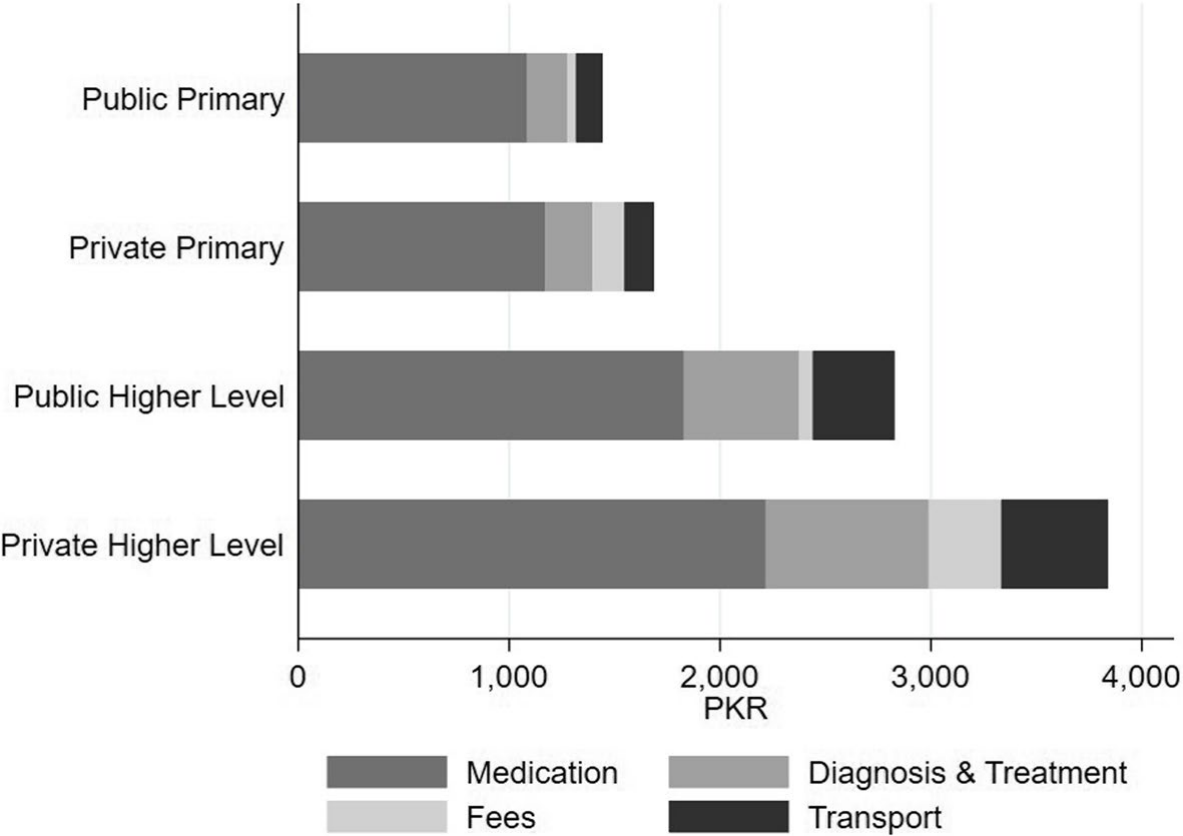
Cost of care by level and component. Bars represent the reported average out-of-pocket expenditures (weighted mean) for the most recent OPD visit of those who reported at least one OPD visit within the past month by facility types in PKR. Different shades of grey indicate the average expenditure composition

We assessed the costs of care for illness episodes across the entire care-seeking pathway, instead of only at the point of utilization (Fig. 7). The average expenditure for an OPD care visit was 1,954 PKR (9.9 EUR, January 2021) across all reported visits, around 5% of average monthly household expenditures, but with a lot of variation across facilities and visits. While visits to primary facilities were more frequent, they were on average less costly than secondary facilities. Exceptionally high expenditures were concentrated in private secondary facilities. Medication expenditures took the highest share among expenditures, followed by diagnosis and treatment, and by transport expenditures. Expenditures were paid out-of-pocket and financed by loans from family or friends and savings. A non-negligible share of our sample also reported to increase the number of jobs or working hours, asked for donations from family, friends, or neighbours, or even reduced consumption to finance the health expenditures. Poor people donated and fundraised for their community members when needed and participants mentioned taking medicines on credit from medical stores. People also mentioned requesting their physician to prescribe less medication to save their costs.

FGD participants mentioned indirect expenditures like accommodation costs if the facility was out of town, food during waiting hours, and informal payments to get medicines from limited stocks, earlier appointments, and quicker diagnostic results.

*‘They are good to those who pay them some money. Their tests take no time*,* also in OPD*,* their turn comes first.’* (Male, Kohat).

Supply-side interviews mentioned physicians deliberately writing shorter prescriptions if they observed that the patient was poor. All interviewed physicians recognized the hardships of the poorest patients in meeting health needs; public facilities reported that they exempted a small number of patients each day, one used donation to buy medicines for the poor, and a private facility physician said that they set up free medical camps sporadically.

#### Healthcare consequences

In the last stage of a care seeking, we consider experiences of care seeking including perceived technical quality and interpersonal interactions.

In public facilities, FGD respondents reported substandard technical quality: medicines were almost never available, and people were asked to bring their own equipment such as thermometers and bandages. Physicians, mentioned that some public primary health centers in KP may not even have the most basic equipment like blood pressure monitors and glucometers. In Chitral, none of the public facilities had a Computed Tomography (CT) or Magnetic Resonance Imaging (MRI) machine. Physicians at public facilities mentioned requesting relevant authorities to improve the situation, but the bureaucratic processes took long.

*‘In my opinion we cannot give 100% services to people. I can say about 50/50 as our hospital is not fully equipped. Our human resource has always been less. We have a deficiency of doctors*,* staff nurses. Lab is also overburdened. Workload is high in our hospital.’* (Public secondary facility, Mardan).

Respondents from private facilities on the other hand, mentioned better laboratories, diagnostics, and pharmacies but with large variations and people reported to visiting laboratories in the market for specialized tests. FGD respondents also experienced slow diagnoses and having to travel to other cities to receive care and diagnostic tests.

Physicians across all facility types were rated well, and people saw them as helpful and knowledgeable, yet private sector physicians were reported to give more attention, time, and be friendlier. Other facility staff such as nurses and receptionists received predominantly negative impressions and were accused of mistreating people based on their socio-economic status.

People’s inability to adhere to the care regimen prescribed to them emerged as a strong theme in the FGD’s: medication was not or only partially purchased, referrals and follow-up instructions were not always followed, at perceived recovery treatment could be given up immediately, and a special diet suited to health conditions could not be arranged, due to lack of finances.

*‘The main reason why we do not have any left money for medicines is because we pay some for ultrasound*,* some in consultation fees*,* and some in lab tests. Since we do not have money*,* we come back home and keep the OPD slip on one side; we are helpless we do not have any other option.*’ (Female, Malakand).

Physicians also reported non-adherence to care among the poorest patients. Very few, however, mentioned measures taken to counter non-adherence mostly in the form of prescribing fewer medications when requested.

## Discussion

As efforts emerge to strengthen PHC systems across the world, complementary knowledge of the health needs of vulnerable populations is crucial to design people-centred initiatives. We offer insights from the poorest population segment in the KP province, Pakistan to add to this body of knowledge. Our results show that poor people’s basic health needs remained unmet, and in cases where they may be considered met, people incurred significant financial and systemic barriers. Gaps in approachability (providing adequate information about navigating the health system), and availability and accommodation (availability of necessary drugs and equipment) made the supply side unable to fully meet the health needs of the poor. On the other hand, people found it difficult to travel to the care that they would prefer (ability to reach) and adhere to prescriptions (ability to engage) leading to unmet needs. We found that out-of-pocket expenditures were a major deterring factor, as expected and shown in other settings [17, 23], yet they were not the only determinant of unmet need. Problematizing simplistic conceptualizations of health needs, we argue that unmet needs are multifaceted; they can exist despite utilization of care. We observed a negative perception of the health system among the poorest population in KP. Previous studies focusing on public trust in health systems have shown that vulnerable populations such as minority races, physically disabled persons, and refugees have lower trust in the public health system, and recommend explicit focus on integration [50–52]. Leveraging trust through policies to strengthen PHC systems could be an opportunity that serves the long-term goals of greater healthcare utilization, trust in public systems, and broader social solidarity [53, 54]. Better staff training [55] and allocating resources for outreach and communication programs could improve the impression of health facilities among the poorest population, invite people to visit facilities for their health needs without delays, and support them in navigating the health system. Complementary programs in the local contexts should be integrated with UHC policies to this end, such as the Primary Care Management Committees in KP, which engage local representation in healthcare decision-making [56].

At the level of seeking healthcare, we observed the intersectional role of poverty with age and gender in determining unmet needs. The differences in underlying factors of unmet need among men and women, reinforce the importance of reflecting on disparate gender effects when studying unmet needs among vulnerable groups. Our findings are consistent with a study conducted by Panezai et al.. in a rural Balochistan, Pakistan, where utilized and forgone BHU care was similar for both sexes, yet factors influencing utilization were different [27]. Other studies on unmet need across various settings also confirm that men and women’s unmet needs are due to different underlying causes [17, 57] therefore it is important to explore, beyond numbers, how access can be improved. Calls to explore the effect of gender in UHC policies have been raised [58], which can be extended to PHC systems as well: while conceptually PHC systems aim to be accessible to all, a gender-blind approach can exacerbate the differences among men and women’s ability to seek and reach the healthcare they need. Considerations of protecting the health of middle-aged people in SHP programs can be done through lowering opportunity costs by offering compensation for daily wages during healthcare facility visits. This is important as middle-aged people are typically seen as independent and empowered enough to meet their needs which was not the case among our respondents.

More broadly, our results have policy implications in that they highlight that SHP is an important tool for advancing UHC for improving PHC but is incomplete without investments made to the overall health system including towards quality and service delivery improvement at public facilities. Moreover, health needs are not limited to the health sector alone and require action from the entire society and the role of multiple administrative sectors. Engaging with different sectors inside and outside of the health system as evident from our results, must go hand in hand with financial protection offered by SHP programs, to improve PHC access for the poor.

Reflecting on the use of Levesque’s framework of access to care, we observed that the stage-by-stage care seeking pathway, while conceptually sound for the purpose of this study, does not reflect the reality for our respondents. As people were unable to complete their care, they found themselves sent back to previous stages a few times, until they were able to meet their needs. Another shortcoming of the framework we observed was the unified idea of a ‘health system’, interacting with the population. In reality, KP’s health system is a combination of providers, some able to meet their needs better than others.

### Methodological considerations

The study has potential limitations. Our quantitative results are an upper bound for monthly health care utilization because we expect some recall bias, i.e., over-reporting due to recall of OPD visits that lie outside the relevant month. It is important to note that we could distinguish between initial and follow-up visits within the same illness episode and to several facility types thus we counted them as separate visits. Moreover, self-assessment of health needs can only capture the perceived needs i.e., health needs not recognized by respondents, could not possibly become a part of our data. Second, while we took utmost care to draw a representative sample from within the selected study districts, remoteness and safety concerns led us to avoid certain neighbourhoods. Thirdly, facility experience could not be captured in our survey as it was conducted at the household level as opposed to a facility exit interview, which would be more suitable to shed light on quality of care experienced. We also acknowledge a power gap between the poorest population and our data collection team, which may have influenced responses despite efforts made to build rapport. To offset these limitations, we reinstate the strengths of our study in its mixed methods design eliciting broad patterns, as well as deeper reflections from our respondents. We tried to ensure cultural sensitivity by working with local researchers to guide us and collecting data in local languages. We conducted regular debriefings between field researchers and the rest of the team allowing for emergent considerations in the research design. Using unmet needs across an access pathway to elicit gaps in the health system offered us a very broad yet patient centred lens to judge the system and make recommendations. Future research analysing objective assessments such as facility surveys would complement this study to establish the capacity of the system to meet health needs.

## Conclusions

The poorest population in KP province, Pakistan reported unmet PHC needs. The needs that were met came with considerable barriers, including but not limited to the affordability of care. Our findings highlight the essential role that awareness plays in the health seeking process, suggesting the need to invest in facility outreach and information programs to ensure that people feel welcome to access care and can be supported to navigate the health system. Having recognised that limited resource provision at facilities contributes to people’s unmet needs, we further recommend that investments are made to improve health facility infrastructure and provision of essential products and services proximate to those with higher needs, to improve overall service quality.

## Supplementary Information

Additional file 1.

Additional file 2.

Additional file 3.

Additional file 4.

## Data Availability

The data that support the findings of this study are available from the corresponding author, M.S., only in anonymized form, and upon reasonable request.

## Abbreviations

BISP: Benazir Income Support Programme
FGD: Focus Group Discussion
IDI: In-depth Interview
KP: Khyber Pakhtunkhwa
LMIC: Low-and-Middle Income Countries
PHC: Primary Healthcare
SHP: Social Health Protection
SHPI: II-Social Health Protection Initiative phase-II
UC: Union Council
UHC: Universal Health Coverage

## Footnotes

1 We follow the program’s definition of core family members that are covered under one beneficiary card: main insurance cardholder (household head), spouse and unmarried children who live in the same household.
