# Supplementary material for: Un/met: a mixed-methods study on primary healthcare needs of the poorest population in Khyber Pakhtunkhwa province, Pakistan
Source: Int J Equity Health. 2024 Sep 23;23:190. doi: 10.1186/s12939-024-02274-5 (PMC11421121; doi:10.1186/s12939-024-02274-5)
Supplement: Supplementary file 1 — Additional file 1. [file 12939_2024_2274_MOESM1_ESM.docx]

**Additional File 1: Qualitative Guides**

**Part 1. Focus Group Discussion Guide**

**Questions**

1. What do you think of the health status in your community?

*Which illnesses are common?*

*How frequently do people get ill?*

1. How do you decide if it is required that you see a professional or seek treatment for your symptoms?
2. Does anyone of you or your family members have diseases that last long like diabetes, hypertension, asthma, epilepsy, heart diseases or etc? Which diseases?

*How did you or they find out? Can you share the whole story?*

*How is care seeking different for you/ these persons? (considering the repetitive visits ,
in terms of finances, social support, medication side effects etc.)*

**Questions 4-13 can be asked in any order, if already covered, they can be skipped.**

**Theme: Choice of facility**

1. Which factors are important to be considered when choosing a facility?

*How do you choose between government and private facilities?*

*How is the hospital where you and your family normally go to? Which facilities does it have (waiting area, pharmacy, sehat card empanelment...)? Which illnesses are treated there?*

*Where do you go for long lasting illness? Where do you go for small/simple illnesses? Why?*

**Theme: Facility experience**

1. During your last visits to the health facility you visited, how was your experience of the quality of health care services provided?

*Do facilities have what you need to care for the illnesses you present with? (medicines, laboratory, bandages and disposable syringes, bp monitor, blood sugar monitors etc.)*

*What was missing?*

*If something is not available, where else are you expected to go (pharmacy, lab etc.)? How far are they? (How expensive?)*

*What do you think about waiting times? (Too long? Just right?) Are waiting rooms available?*

*How was the interaction with staff (receptionists, nurses etc.)? (Are they welcoming, rude, overburdened…?)*

**Theme: Patient-Physician relationship**

1. During your last visits to the health facility you visited, how was the attitude of the doctor?

*Do doctors explain the treatment regimen properly?*

*Do doctors listen to your complaints and respond to your questions to satisfaction?*

**Theme: Referral**

1. Have you ever been referred from one doctor to another? Was your convenience/ease taken into consideration?

If any, what problems did you face in accessing the referred practitioner?

*Do government doctors sometimes ask patients to come to their private practice
 instead? Why?*

**Theme: Location, travelling and timing**

1. Do you think that the location of your provider/facility is convenient to you, in relation to where you live? (cost, distance, means of transport)

*Who goes to the facility with you?*

*How do chronic patients manage these costs in repeated visits?*

*What about opening times or OPD days? (also considering travel time to facility)*

*Do you have to make appointments (or can you walk-in)? Is it easy to make an appointment?*

**Theme: Fees and finances**

1. What do you think of doctors’ fees?

*Are all services paid for, separately? Which ones? (consultation, medication etc.)*

*Which services do you find most difficult to access especially due to fees?*

*What could be the reasons to prefer more expensive services when cheaper alternatives*

*are available? (e.g. for better quality, trust, close to home etc.)*

*How do patients manage these costs if they are expected to visit frequently?*

*Which illnesses (chronic vs. acute) are considered most expensive for OPD? Why?*

*(Consultation, diagnostics, tests, medicines)*

*If someone needs equipment to support treatment at home like BP monitor, nebulizer, crutches etc., how do they get it?*

1. The last time that you or a family member became ill, how did your household manage to pay for health care services?

*In your opinion, what can someone do if they do not have enough money to pay*

*for healthcare?*

*Which costs do you have to keep in mind for treatment? (travelling costs and fees -
also probe on lab tests, medicines, equipment like bandages, crutches etc.)*

**Theme: Social support**

1. How supportive is your family and community regarding health seeking if one has a chronic condition? (financially, socially. giving time and company, understanding their problems, getting annoyed)

**Theme: Alternative therapies**

1. Are there any hakeem or homeopathic practitioners that you visit? How does your experience with them compare to that with ‘allopathic’ practitioners? (*In terms of…cost, distance, interpersonal communication, effectiveness of treatment, trust)*

**Closing**

1. Imagine you are a health care provider- what do you think would be in your power to change to overcome current barriers to access you describe and provide services that meet your needs? What would be beyond the health provider’s control and would need to be changed at higher level?

[Thank you note at the end]

Questions by Lévesque’s framework dimensions

| **Dimensions of access** | **Question** |
| --- | --- |
| Ability to perceive | 1, 2, 3 |
| Ability to seek | 3, 4, 5 |
| Ability to reach | 4, 7, 8, 11 |
| Ability to pay | 4, 9, 10 |
| Ability to engage | 4, 5, 6 |

**Part 2. In-Depth Interview Guide**

**Questions**

1. Ice breaker: Could you please re-introduce yourself and your facility for the record?
   [do not transcribe any details except the level of the hospital as primary, secondary, tertiary and, its status either public or private. The only introduction of the physician to be kept in the transcription is gender and specialization]

*Which services does your facility offer? Are they free of charge or paid? If free of charge to the patient, who is covering the cost?*

*How large is it, by number of outpatient consultations per week? (An estimate)*

*Why do you think clients choose your facility?*

*Private only: Where do most of your patients find out about your practice?*

1. What is the general patient profile (socio-economic segment) visiting the OPD at your facility? (How many ultra-poor)

*Where do you patients come from? (geographically)*

*Which conditions do they suffer from?*

*Which conditions are common among the poor? Are they able to afford payment?*

*Have you observed a difference in disease management, especially for chronic conditions, based on socioeconomic background of the patients?*

1. What expectations do clients have of getting treatment at your facility’s OPD? What do you think are their most important needs?

*What are some unrealistic expectations that you have come across and why do you think they exist?*

**Information:**

1. What do you think about the level of health awareness among your clients?

*To what extent do you think that lack of treatment seeking is rooted in lack of illness perception?*

*What do you describe good health as?*

*Do you think your patients views on the idea of good health be the same? What is good health for them?*

**Local norms and practices:**

1. How do the services currently being offered at your facility ensure clients’ comfort regarding religious or cultural values? (For instance, do you have female staff? Do you allow a companion to accompany women? Even in times of covid?)
2. How do you view alternative health practices? (Hakeem, homeopathic, damm darood etc.) Why do people go them?

*Gher doctori elaj ke baare mein aapka kya khayal hai, yani homeopathic, hakeemi elaj waghaira? Log kyun unke pass jate hain?*

**Access:**

1. What role does geographical proximity and timing, play in access to care? (such as location, opening hours/number of OPD days)

*Have you received complaints regarding opening hours, number of OPD days etc. from
your clients? What did your facility do about them?*

*What are some of the reasons poor people may not be able to get treatment even when they*

*are able to physically approach care?*

1. *Can you please describe the referral mechanism in your area? (Who refers patients to you? To whom do you refer patients?)*

**Finances:**

1. How significant is the problem of the clients’ inability to pay, in your experience, for healthcare that they need?

*In your experience which expenses do patients have to consider while seeking treatment (medicines, diagnostics, transport, other consumables etc.)*

*How is this different for chronic condition patients?*

*Private: Do the fee packages vary by SES? Is it possible to exempt the poor? Public: Which policies are there to exempt the poor for diagnostic services and medicines etc.?*

**Resources:**

1. Are providers able to discuss treatments and regimens reasonably with the clients based on the time and resources they have? (Why, why not?)
2. How well equipped are health facilities in KP, in general? Is it sufficient to meet the needs of the people? (in terms of staff, diagnostic, drugs etc.)

*What is the availability status of drugs and equipment to treat the conditions you diagnose
in your facility? If they are not available, why, and under which circumstances?*

**SHPI:**

1. What do you think of the SHPI Scheme/Sehat Card Plus scheme? Is it really helpful in catering to the needs of the poorest?

*Is your facility a part of the SHPI?*

*How would you feel about the introduction of a similar scheme for outpatient care?*

*Would you like to be empaneled for it? Why/ why not?*

**Closing:**

1. A slightly different question and with all due respect: Suppose you were to trade places with one of your poorest patients, what do you think, based on the lay knowledge, your greatest health seeking struggles would be? I would like to see if I’m missing any fine details in understanding the client, which you as a doctor can observe.

*Questions by Levesque’s framework dimensions*

| **Dimensions of access** | **Question** |
| --- | --- |
| Broad | 1, 2, 3, 4, 13 |
| Approachability | 5 |
| Acceptability | 6, 7 |
| Availability and accommodation | 8, 9 |
| Affordability | 10 |
| Appropriateness | 11, 12 |
