# Supplementary material for: Un/met: a mixed-methods study on primary healthcare needs of the poorest population in Khyber Pakhtunkhwa province, Pakistan
Source: Int J Equity Health. 2024 Sep 23;23:190. doi: 10.1186/s12939-024-02274-5 (PMC11421121; doi:10.1186/s12939-024-02274-5)
Supplement: Supplementary file 2 — Additional file 2. [file 12939_2024_2274_MOESM2_ESM.docx]

**Additional file 2: Quantitative Sampling Strategy**

Out of the eligible households (52,703) who live in the accessible UCs, the sample was selected in three stages as follows. The procedure is described for households

1. UC-level: We randomly drew four accessible union councils per district.
2. Village-level: Within the UCs, we excluded all villages with less than 40 poor households on our sampling list and those that are farther than one hour by car away from the closest RHC.^[[1]](#footnote-1)^ Out of the resulting list of villages per district, half was again randomly selected to do a discrete choice experiment (not included in this manuscript) in addition to the household survey. This yielded up to two villages per UC and a total of 22 villages (Chitral: 7, Kohat: 7, Malakand: 3, Mardan: 5).
3. Household-level: If there were two villages in a UC, we sampled 40 households from each. If there was only one village, we sampled 80 households still reach the number of 80 households per UC. Re-sampling of some villages became necessary for Kohat (within and outside the district) due to a worsened security situation. We drew 4 times as many households from the sampling frame as we aimed to interview as the list was old and imprecise so we expected to on the one hand not find many households, but also exclude some due to death or migration.

1. For district Chitral, we included all accessible UCs and the respective villages to reach the desired sample size. [↑](#footnote-ref-1)
