# Supplementary material for: Un/met: a mixed-methods study on primary healthcare needs of the poorest population in Khyber Pakhtunkhwa province, Pakistan
Source: Int J Equity Health. 2024 Sep 23;23:190. doi: 10.1186/s12939-024-02274-5 (PMC11421121; doi:10.1186/s12939-024-02274-5)
Supplement: Supplementary file 3 — Additional file 3. [file 12939_2024_2274_MOESM3_ESM.docx]

Additional file 3: Quantitative results

**Table 1: Trust in people and medical professionals**

|  | Completely | Somewhat | Not very much | Not at all |
| --- | --- | --- | --- | --- |
| Trust in people | 0.41 | 0.32 | 0.12 | 0.15 |
| Trust in medical practitioners | 0.59 | 0.33 | 0.03 | 0.06 |

Table 2 Regression explaining reported outpatient care need by proxies of expected need (age, health condition, gender, district) and desire to seek care (wealth, distance to health facility)

|  | (1) | (2) | (3) |
| --- | --- | --- | --- |
|  | All household members | Adults only | Decisionmakers only |
| Age group (ref: 15-50 years) |  |  |  |
| Below 15 years | 2.526^***^ |  |  |
|  | [1.870,3.413] |  |  |
|  |  |  |  |
| Above 50 years | 2.827^***^ | 2.627^***^ | 2.316^***^ |
|  | [1.952,4.093] | [1.783,3.871] | [1.442,3.720] |
|  |  |  |  |
| Health condition | 1.112 | 1.106 | 1.189 |
| (Scale 1=good to 5=poor) | [0.937,1.321] | [0.915,1.338] | [0.966,1.464] |
|  |  |  |  |
| Gender (female=1) | 1.317^**^ | 2.036^***^ | 1.462^**^ |
|  | [1.065,1.628] | [1.498,2.767] | [1.037,2.062] |
|  |  |  |  |
| Asset index | 1.365^***^ | 1.471^***^ | 1.703^***^ |
|  | [1.182,1.576] | [1.240,1.745] | [1.405,2.064] |
|  |  |  |  |
| Distance to closest rural health center (km) | 0.971 | 0.962^*^ | 0.953^*^ |
|  | [0.937,1.006] | [0.926,1.000] | [0.908,1.000] |
|  |  |  |  |
| District (ref=Chitral) |  |  |  |
| Kohat | 1.799^**^ | 1.956^***^ | 2.772^***^ |
|  | [1.126,2.873] | [1.179,3.244] | [1.521,5.050] |
|  |  |  |  |
| Malakand | 2.871^***^ | 4.489^***^ | 10.60^***^ |
|  | [2.004,4.113] | [2.858,7.051] | [5.831,19.28] |
|  |  |  |  |
| Mardan | 2.031^***^ | 3.106^***^ | 3.086^***^ |
|  | [1.495,2.760] | [2.181,4.422] | [1.932,4.931] |
| Obs. | 3835 | 2277 | 1375 |

Odds ratios from logit regression with binary dependent variable: household member had any health need during the previous month, for which s/he considered or sought outpatient treatment; each column has a different sample indicating a higher level of own decision-making power: column (1) covers all household members, (2) all members over the age of 18. (3) only member for whom the main respondent reported standard errors clustered at household level; 95% confidence intervals in brackets; ^*^ *p* < 0.10, ^**^ *p* < 0.05, ^***^ *p* < 0.01

**Table 3: Gender difference in OPD transportation cost**

|  | Men | Women | Difference | p-value |
| --- | --- | --- | --- | --- |
| OPD transport cost | 191.63 | 223.16 | 31.53 | 0.18 |

**Table 4: Gender difference in health decision-making**

|  | Adult men | Adult women | Difference | p-value |
| --- | --- | --- | --- | --- |
| Share health decision-maker | 0.71 | 0.40 | -0.31 | 0.00 |

**Table 5: Age difference in OPD transportation cost**

|  | Young | Old | Difference | p-value |
| --- | --- | --- | --- | --- |
| OPD transport cost | 182.91 | 308.96 | 126.05 | 0.00 |

**Table 6: Chronic care need and OPD transportation cost**

|  | No chronic | Chronic | Difference | p-value |
| --- | --- | --- | --- | --- |
| OPD transport cost | 191.52 | 286.93 | 95.41 | 0.01 |
