# Supplementary material for: Un/met: a mixed-methods study on primary healthcare needs of the poorest population in Khyber Pakhtunkhwa province, Pakistan
Source: Int J Equity Health. 2024 Sep 23;23:190. doi: 10.1186/s12939-024-02274-5 (PMC11421121; doi:10.1186/s12939-024-02274-5)
Supplement: Supplementary file 4 — Additional file 4. [file 12939_2024_2274_MOESM4_ESM.doc]

Additional File 4: Household survey

Content

[0 Preparation 2](#__RefHeading___Toc100828965)

[1 Household and Personal Information 4](#__RefHeading___Toc100828966)

[1.1 Household information 5](#__RefHeading___Toc100828967)

[1.2 Personal information & attitudes 7](#__RefHeading___Toc100828968)

[2 Economic Condition 10](#__RefHeading___Toc100828969)

[2.1 Employment 10](#__RefHeading___Toc100828970)

[2.2 Housing situation / poverty 12](#__RefHeading___Toc100828971)

[2.3 Assets 12](#__RefHeading___Toc100828973)

[2.4. Expenditures 14](#__RefHeading___Toc100828974)

[3. Insurance Awareness and Coverage 16](#__RefHeading___Toc100828975)

[3.3. Sehat Sahulat Program Beneficiaries 18](#__RefHeading___Toc100828976)

[4. Health History and Costs 23](#__RefHeading___Toc100828977)

[4.1. General situation and risk perception 23](#__RefHeading___Toc100828978)

[4.2. Childbirth 26](#__RefHeading___Toc100828979)

[4.3. Health Care Utilization 28](#__RefHeading___Toc100828980)

[4.4. Inpatient Treatment 30](#__RefHeading___Toc100828981)

[4.5. Outpatient Treatment 38](#__RefHeading___Toc100828982)

[4.6. Neglected Health Care 43](#__RefHeading___Toc100828983)

[5. End of Interview Page 49](#__RefHeading___Toc100828984)

[6. Enumerator Feedback 50](#__RefHeading___Toc100828985)

# Preparation

[Tablet] record start time, device ID, assign unique form ID

- 1. Interviewer name [single select out of list of all interviewer names]

[next page]

- 1. To determine household to visit, please select your location:

**District** *[drop down fixed menu]***, Union Council** *[drop down fixed menu]***, Tehsil** *[drop down fixed menu]***, Revenue Village** *[drop down fixed menu]*

[next page]

[comment] Please find the following household:

[Tablet: automatically display name of cardholder / age / adress / maybe phone number of a randomly selected household in this village]

[Tablet: save this household’s ID as id_survey without displaying it]

- 1. [single select] Was the household identified?

*1- Yes, via address alone* [continue]

*2- Yes, via asking around the village* [continue]

*3- Yes, via phone contact* [continue]

*4- No, house not inhabited* [end -> but still keep coordinates field]

*5- No, moved away* [end -> but still keep coordinates field]

*6- No, no one knows household* [end -> but still keep coordinates field]

*7- No, other* [add free text field to specify]

- 1. [if yes; single select] Did the household agree to proceed with the consent part?

*1- Yes* [continue]

*2- No, refused* [specify in 0.5]

*3- No, appointment made for another time* [end]

- 1. [if no, refused; free text] Why? [end -> but still keep coordinates field]
  2. Please ask to talk to the listed person. Was this successful?

*1- Yes [continue with 0.6.1]
0- No [continue with 0.6.3]*

- - 1. [if yes] Check CNIC of main card cardholder: “Is this your CNIC?”

[Tablet: display CNIC of listed person]

*1- Yes [continue with 0.6.2]
0- No* [end of interview]

99- don’t know *[continue with 0.6.2]*

- - 1. [If yes | don’t know in 0.6.1] Is the listed person able to conduct the interview? (Instruction: if the age of the possible respondent is over 65, please ask whether a younger household member is available, please also make sure that the respondent can hear and speak one of the interview languages sufficiently well to follow this interview and select the respective answer below)

*1- Yes, now [continue with introduction & consent]*

*2- Yes, over 65 but insists to do the interview him/herself [continue with introduction & consent]*

*3- No, over 65 years*

*4- No, has hearing impairment*

*5- No, not willing to be interviewed*

*6- No, does not speak any of the interview languages.*

*7- No, other, specify: _________________*

- - 1. [If no in 0.6] Why could the listed person not be reached?

*1- Not at home*

*2- At home, but not available for interview*

*3- Not member of household anymore (e.g. died, left home after marriage)* [end of interview]

- - 1. [If 0.6.3 < 3] Is the listed person’s spouse available?
       *1- Yes* [continue with introduction & consent]
       *0- No*
    2. [If no in 0.6.4] Is any other adult (but unmarried) core family member of the listed person available? *1- Yes, listed person’s child* [continue with introduction & consent]
       *0- No* [end of interview]

[next page]

**INFORMED CONSENT**

Thank you for agreeing to learn more about our study.

To interviewer: Please go through the information sheet with the participant.

Please acknowledge the following points after they were completed:

[checkbox] The information sheet was read and explained.

[checkbox] The respondent has no further questions.

[checkbox] The information sheet and consent form was handed to the respondent.

- 1. Does the respondent agree to participate in the survey?

*1- Yes* [proceed]

*0- No*

- 1. [If yes] Please sign the consent form, hand one copy to the respondent, keep the other one and take a picture of the signed form:

[Tablet: insert image field to take a picture and save the file name in the dataset]

# Household and Personal Information

[timestamp]

[tablet: include option “refused to answer” to all questions from here on, for all questions an answer should be mandatory]

(comment: Instruction: In case the interview is stopped before the end, please select “refused to answer” for all remaining questions and specify the reason for stopping the interview in the comments field in the end)

## Household information

**[Introductory Page 1: Household Roster]**

[comment] I would like to talk with you about your household. As the household I define all the people living in the same place and sharing expenditures for food. I would like to talk about all the household members that are currently present or left for short period of time (less than 6 months). Please give the names of all members of your household. Start with yourself.

- - 1. Please name the members of your household who usually live here. Do not list guests or visitors:

[If the respondent is unwilling to name the HH member, the relation to the respondent shall be entered instead. E.g. “your son”]

[Tablet: calculate number of names entered and save this variable without displaying it directly]

[Tablet: display] You have entered [number of names] names: [list all names]. Is this correct? [If the answer if no, go back to the list and modify it until it is correct.]

[Tablet: round 1 is for the respondent, so replace [name X] with you / your.]

| - - 1. Is [name X] male or female?   *1- Female*  *0- Male* | - - 1. What is [name X]’s relation to [main cardholder]?   *1- Self [meaning that [name X] is the main cardholder]*  *2- Husband/ Wife*  *3- Son/ Daughter*  *4- Father/Mother*  *5-Brother/Sister*  *6- Grand Son/ Grand Daughter*  *7-Father/Mother in Law*  *8- Brother/Sister of Husband/Wife*  *9- Other Relative*  *10- Not a Relative* | - - 1. How old is [name X] (in years)   [Instruction: If younger than 1 year enter “1”]  [numeric, restrict to 0-100] | - - 1. [if age 16 and older]   Is [name X] responsible for day-to-day decisions about money in your household?  *1- Yes*  *0- No*  *99- Don’t know* | - - 1. [if age 7- 17]   Is [name X] attending school?  *1- Yes*  *0- No*  *99- Don’t know* | - - 1. [if age 7 and older]   What is [name X]‘s highest attainment of formal education?  *1- no formal education (0)*  *2- Some primary school (1-4)*  *3- Completed Primary School (5)*  *4- Some Middle/ High School (6-7)*  *5- Completed Middle/ High School (8)*  *6- Senior Secondary School/ Matric (10)*  *7- Higher Secondary School (12)*  *8- Tertiary education (College or beyond) (>12)*  *99- Don’t know* | - - 1. [if age 15 or older & relation not husband/ wife]   What is [name X]‘s marital status?  *1- never married*  *2- married*  *3- widowed*  *4- divorced*  *5- separated*  *99- Don’t know* |
| --- | --- | --- | --- | --- | --- | --- |

| - - 1. Does [name X] have an own CNIC number [if age is 18 or older]?   *1- Yes*  *0- No*  *99- Don’t know*  [if selected “no” here & yes in 0.6 display error message: You entered that you are speaking to the main cardholder, this person must have a cnic number.] | - - 1. [if yes in 1.1.9]   Please enter [name X]’s CNIC number:  [numbers, restrict to the standardized format of cnic numbers]  *99- don’t know*  *88- refusal*  OR  [Instruction: add 9999999999999 for don’t know and 8888888888888 for refused] | - - 1. In general, would you say [name X]’s health in general is excellent, very good, good, fair, or poor?   *1- excellent*  *2- very good*  *3- good*  *4- fair*  *5- poor*  *99- Don’t know* |  |  |
| --- | --- | --- | --- | --- |

## Personal information & attitudes

[Read]

After talking about your whole household, I now would like to ask you a few questions about yourself. We will start with how you have been feeling during the past two weeks.

| Headline: WHO-5 Well-being Index   - - 1. Please indicate for each of the five statements which is closest to how you have been feeling over the last two weeks, using a scale from 0 to 5, where 0 means you have been feeling like that ”at no time”, 1 means “some of the time”, 2 means less than half of the time”, 3 means “more than half of the time”, 4 means “most of the time” and 5 means “all the time” . Notice that higher numbers mean better well-being.   Over the last two weeks, how often have you felt…? [For 1.2.1.1 until 1.2.1.3.]  Over the last two weeks, how often did you…? [For 1.2.1.4]  Over the last two weeks, how often has your daily life….? [For 1.2.1.5.]   | Over the last two weeks | All the time | Most of the time | More than half of the time | Less than half of the time | Some of the time | At no time | | --- | --- | --- | --- | --- | --- | --- | | - - - 1. …cheerful and in good spirits. | 5 | 4 | 3 | 2 | 1 | 0 | | - - - 1. …calm and relaxed. | 5 | 4 | 3 | 2 | 1 | 0 | | - - - 1. …active and vigorous. | 5 | 4 | 3 | 2 | 1 | 0 | | - - - 1. woke up feeling fresh and rested. | 5 | 4 | 3 | 2 | 1 | 0 | | - - - 1. been filled with things that interest me. | 5 | 4 | 3 | 2 | 1 | 0 | |
| --- | --- | --- | --- | --- | --- | --- | --- | --- | --- | --- | --- | --- | --- | --- | --- | --- | --- | --- | --- | --- | --- | --- | --- | --- | --- | --- | --- | --- | --- | --- | --- | --- | --- | --- | --- | --- | --- | --- | --- | --- | --- | --- |

We just talked about how you felt specifically during the past two weeks. I will now ask you how you feel about risk in general.

| - - 1. How willing or unwilling are you to take risks, using a scale from 0 to 10, where 0 means you are “completely unwilling to take risks” and 10 means you are “very willing to take risks”. You can also use any number between 0 and 10 to indicate where you fall on the scale: | | | | | | | | | | | |
| --- | --- | --- | --- | --- | --- | --- | --- | --- | --- | --- | --- |
| 0 completely unwilling to take risk | 1 | 2 | 3 | 4 | 5  Neither willing nor unwilling | 6 | 7 | 8 | 9 | 10  very willing to take risk | 99  Don’t know |

| - - 1. I‘d like to ask you how much you trust people from various groups. Could you tell me for each whether you trust people from this group completely, somewhat, not very much or not at all? Even if you have had very little or no contact with these groups, please base your answer on your general impression of them.   [single select, repeat for 1.2.3.1 to 1.2.3.4]  1- Trust completely / Poora yakeen  2- Trust somewhat / Lag der yakeen  3- Do not trust very much / Der yakeen pay nakoma  4- do not trust at all / Bilkul pay yakeen na koma  99- Don’t know]   - - - 1. People in general. / Taaso da aam awam pa khabaro se had pooray yakeen kavaee?       2. Medical practitioners. / Taso da doctorano paighamato/khabaro banday sumra yakeen laraee?       3. Civil servants in your area       4. The national Government |
| --- |

# Economic Condition

[timestamp]

## Employment and Income

[READ: We just talked about your willingness to take risk. Every person can get sick and health incidents can lead to financial stress for a household. Therefore, I would now like to understand better what the financial situation of your household looks like. In a first step, I am interested in who in your household does work for pay.~~]~~

| - - 1. Name   [Tablet: only show names of household members that are older than 7.]  [Tablet: when talking about a child (7-17): “work” might for example mean to work on the family farm from time to time or to help out in a family business.  Below the age of the person is displayed.] | - - 1. Did [name X] do any work for pay, profit or family gain in the last 12 months, i.e. since [display month and year 12 months prior to the survey date]?   *1- Yes [go to 2.1.3]*  *0- No* [go to 2.1.4]  99-don’t know  88-refused | If Yes in Error: Reference source not found   - - 1. What is [name X]’s occupation, that is, what kind of work does [name X] mainly do?   [Probe to obtain detailed information on the kind of work [name X] does]  *1-Government employee (Sarkari naukar)*  *2- Contracted private employee (Private naukari)*  *3- Self employed (Khpal karobaar)*  *4- Unemployed (Iss kaar mey nashta)*  *5- Retired (Retired)*  *6- Daily wage worker (Dehaaree maar)*  *7- Other, please specify: ________*  *99- Don’t know* | If No in 2.1.2:   - - 1. What did [name X] do for most of the time over the last 12 months, i.e. since [display month and year 12 months prior to the survey date]?   [single select]  *1- Going to school/ studying / religious studies*  *2- Looking for work*  *3- Retired*  *4- Cannot work (Handicapped or too ill to work currently)*  *5- Housework / Child care/ Family duties*  *7- Other, specify*  *99- Don’t know* | If Yes in 2.1.2 OR 1 in 2.1.4:   - - 1. Du-ring the last four weeks, i.e. [display month and year 4 weeks prior to the survey date], how many days did [name X] miss at school or work because he/she was sick?   Number in days:  _____________  [Instruction: if none, enter 0] | If Yes in 2.1.2 OR 1 in 2.1.4:   - - 1. During the last four weeks, i.e. since [display month and year 4 weeks prior to the survey date], how many days did [name X] miss at school or work because he/she either had to take care of sick family members at home or had to accompany family members to a health facility?   Number in days:  _____________  [Instruction: if none, enter 0] |
| --- | --- | --- | --- | --- | --- |

## Housing situation / poverty

[READ: To get to know your living situation better (and assess the impacts this has on health) I would now like to know more about your housing situation.

[Instruction. (Do not read) The main idea is to assess the living standards of the respondent. Since this section contains a lot of questions, refrain from asking obvious questions. For example: If the bulb is on, then it is obvious that there is electricity.]

| - - 1. How many rooms does your house have?   [enter 99 for don’t know, 88 for refused]  [integer numbers, Check: >0 &<50, 99, 88] |
| --- |
| - - 1. What is the principal type of toilet facility used by your household? [don’t read options]   *1- Flush connected to a public sewerage, to a pit or to an open drain*  *2- Dry raised latrine or dry pit latrine*  *3- There is no toilet in the household* |

### *2.2.1*

## Assets

[READ: To learn even more about your living condition (and relate this to health), I would now like to know about specific items and assets your household possesses. I will read different items to you and I would like you to tell me whether the respective items are present in your household.]

- - 1. HOUSEHOLD ASSETS

| Household Assets |  |
| --- | --- |
|  | Yes / no |
| - - - 1. LIVING CONDITIONS   [Introduction page]  [READ]  I would like to start with items that you might use in your everyday life.  Is there …. In your household? |  |
| TV |  |
| A Radio |  |
| Simple mobile telephone |  |
| Smartphone |  |
| Fridge / freezer |  |
| cooking stove or cooking range |  |
| microwave oven |  |
| Washing machine |  |
| A room cooler /an air conditioner? |  |
| A computer/ laptop? |  |
|  |  |
| - - - 1. TRANSPORTATION   [Introduction page]  [Read]  In a next step, I am interested in which kind of transportation vehicles the members of your household own.  Does anybody own … ? | Yes / no |
| Bicycle/ Tricycle |  |
| Motorcycle or motor scooter |  |
| Car/ truck/ bus |  |
| Tractor |  |

- - 1. AGRICULTURAL ASSETS

[READ: It might be that some of your household members are engaged in farming or livestock activities to earn income. Apart from that your household might engage in farming or livestock activities in order to produce food for your own consumption. I am now interested in all agricultural activities your household is engaged in.]

|  | [Quantity] |
| --- | --- |
| - - - 1. How much cultivable agricultural land do members of your household own? (in acres) | ______(Acres) |
|  |  |
| - - - 1. Thinking about livestock, how many of the following does your household own? |  |
| Cows |  |
| Bulls/ Buffalo |  |
| Goats / Sheep |  |

## Expenditures

I just asked you about the assets your households owns. Now I would like to talk to you about the expenditures that your household has on a regular basis.

| 2.5.1.A  How much does your household usually spend monthly on…? | | | | | | | [Estimated value in Rs.] | | | | |  | |
| --- | --- | --- | --- | --- | --- | --- | --- | --- | --- | --- | --- | --- | --- |
| Electricity Bill | | | | | | |  | | | | |  | |
| Fuel | | | | | | |  | | | | |  | |
| House Rent | | | | | | |  | | | | |  | |
| Food Expenditures | | | | | | |  | | | | |  | |
| Guest Expenditures | | | | | | |  | | | | |  | |
| Children Expenditures | | | | | | |  | | | | |  | |
| Books Expenditures | | | | | | |  | | | | |  | |
| Clothing | | | | | | |  | | | | |  | |
| Travelling (Within city/town) | | | | | | |  | | | | |  | |
| Milk | | | | | | |  | | | | |  | |
| Amount of installments (of any) | | | | | | |  | | | | |  | |
| Health OPD related expenses | | | | | | |  | | | | |  | |
| Health IPD/Hospitalization | | | | | | |  | | | | |  | |
| Others | | | | | | |  | | | | |  | |
| Total Expenses | | | | | | |  | | | | |  | |
|  | | | | | | | [Check: amount >=0, <1,000,000] | | | | |  | |
| 2.5.1.B  Do the joint household earnings per month usually cover the joint household expenses per month?  1- yes  2- no  99- don’t know  88- refused | | | | | | | | | | | |  | |
| 2.5.1.C On a scale from 0 (completely dissatisfied) to 10 (completely satisfied), how satisfied are you with the financial situation of your household? | | | | | | | | | | | | | |
| 0 completely dissatisfied | 1 | 2 | 3 | 4 | 5  neither satisfied nor dissatisfied | 6 | | 7 | 8 | 9 | 10  completely satisfied | | 99  Don’t know |

# Insurance Awareness and Coverage

[timestamp]

[Read: In the following sections, we want to talk about health and health expenditure in particular. Paying for health care can be difficult at times.]

| - - 1. On a scale from 1 (I strongly disagree) to 5 (I strongly agree), how do you think about the following statement:  During the past 12 months, finding the money to pay for health care of my household members has been difficult.   *1- I strongly disagree 2- I disagree.*  *3- I neither agree nor disagree. 4- I agree. 5- I strongly agree.*  *88- Refused* |
| --- |

[Read: When it comes to health expenditures, paying it from savings or credit is common. But there are also other ways:]

| - - 1. Have you heard of a program/way that can reduce the burden of health costs, i.e. that some (or all) of the health services are paid for directly at the facility or you can get reimbursed for costs later on?   *1- Yes* [proceed to 3.0.3] *0- No* [proceed to 3.0.4] |
| --- |
| If yes in 3.0.2  ------------------   - - 1. Is any member of your household enrolled in such a program/ can any of your household members use such a way to pay for or get reimbursement for health expenses?   *1- Yes* [proceed to 3.0.3.1]  *0- No* [proceed to 3.0.4] |
| If yes in 3.0.3  ------------------   - - - 1. Which health insurance is it? [don’t read options]   *1- Sehat Sahulat Program/Sehat Card* [proceed to 3.1.1]  *2- Any private health insurance (e.g. Jubilee, Adamjee)*  *3- Other public (e.g. SLIC, BISP)*  *4- Other, specify: __________________*  *99- Don’t know* |

| [If no in 3.0.2 OR no in 3.0.3 OR if in 3.0.3.1 “*1*” was NOT selected i.e. if Sehat Sahulat Program was not mentioned by respondent]   - - 1. Have you heard of the Sehat Card/ Sehat Sahulat Program?   *1- Yes* [proceed to 3.0.4.1]  *0- No* [proceed directly to 4.1] |
| --- |
| If yes in 3.0.4  -----------------   - - - 1. According to your knowledge, does the Sehat Card/ Sehat Sahulat Program cover the health care expenses of any of your household members?   *1- Yes* [proceed to 3.1.1]  *0- No* [proceed to 3.0.4.1.1]  *99-* *Don’t know* [proceed to 3.0.4.1.2] |
| If no in 3.0.4.1  -----------------   - - - - 1. Why does the Sehat Card/ Sehat Sahulat Program not cover health expenses of any of your household members?   *1- Not eligible for the program*  *2- Did not enroll for the program* [proceed to 3.0.4.1.1.1]  *3- Card expired*  *4- Other reason, please specify: _________________________________________________________________________*  [proceed directly to 4.1 if 1 or 3 or 4 in 3.0.4.1.1]  If “*2*” in 3.0.4.1.13.0.4.1.1  --------------------  Why did your household not enroll in the program? [don’t read options]  *1- Too expensive*  *2- No access/opportunity to enroll*  *3- No need*  *4- Low value: services not accessible*  *5- Low value: services bad*  *6- Low value: don’t trust insurance*  *7- Cultural/ religious reasons*  *8- Don’t know enough/ too little information*  *99- Don’t know*  [proceed directly to 4.1] |
| If “*99*” in 3.0.4.1  ----------------------   - - - - 1. Why are you unsure whether the Sehat Card/ Sehat Sahulat Program cover health expenses of any of your household members?   *1- Unsure about eligibility*  *2- Unsure about enrolment*  *3- No-one provided information regarding the Sehat Card/ Sehat Sahulat Program*  *4- Other reason, please specify: __________________________________________*  [proceed directly to 4.1] |

## Sehat Sahulat Program Beneficiaries

[3.1 ONLY for respondents where household enrolled in Sehat Sahulat program and respondent is aware of it, i.e. 3.0.3.1 = 1]

| - - 1. Who in your household is covered by the Sehat Card/Sehat Sahulat Program?   *1- All household members*  *2- Only some of the members* [proceed to 3.1.1.1]  If “2” in 3.1.1  -----------------  [Tablet: Show household roster and ask the following question for each household member]   | [Name] | - - - 1. Is [name X] covered by the Sehat Card/ Sehat Sahulat Program?   *1- Yes*  *0- No*  *99- Don’t know* | | --- | --- | |
| --- | --- | --- |
| Headline: Covered Incidents   - - 1. Which kinds of incidents are covered by Sehat Card/Sehat Sahulat Program? [Instruction: Read Options]   *1- Only Inpatient Treatment*  *2- Only Outpatient Treatment (OPD)*  *3- Inpatient and Outpatient Treatment (OPD)*  *99- Don’t know* [don’t read] |
| Headline: Covered Providers   - - 1. Where can you go to receive treatment with Sehat Card/Sehat Sahulat Program? Can you go to receive treatment with the Sehat Card/Sehat Sahulat Program at a….? Can you go to any, some or none of….? [each row is a single select question]   [Instruction: Give examples for the categories if they are not understood. Deduce from Answer and Probe for the specific options that are mentioned]   |  | Any = 1 | Some = 2 | None = 3 | Don’t know =99 | | --- | --- | --- | --- | --- | | Public primary care facility (e.g. BHU, RHC…) |  |  |  |  | | Private primary care facility  (e.g. private doctors/GPs, private clinics…) |  |  |  |  | | Public secondary care facility  (e.g. DHQ, THQ, specialized public hospitals….) |  |  |  |  | | Private secondary care facility  (e.g. private hospitals…) |  |  |  |  | |
| Headline: Coverage Limit   - - 1. Is there a limit to the covered health care expenses (per person per year)?   *1- Yes [proceed to 3.1.4.1]*  *0- No*  *99- Don’t know*  [if yes in 3.1.4]  -------------------   - - - 1. What is the limit (in PKR)? [integer numbers; >0 & < 1,000,000 ]   _____________ PKR |
| [Read: In the following, we are interested in your experience with the Sehat Card/Sehat Sahulat Program.]   - - 1. Has your household ever used the Sehat card/ Sehat Sahulat Program?   *1- Yes* [proceed to 3.1.5.2]  *0- No* [proceed to 3.1.5.1]  *99- Don’t know* |
| If yes in 3.1.5  How many times did your household use the Sehat card / Sehat Sahulat Program? [integer, 99 for don’t know] |
| if no in 3.1.5  ------------------   - - - 1. Why has no one in your household ever used the Sehat card / Sehat Sahulat Program? [multiple select]   *1- No need*  *2- Did not know how to use it.*  *3- No empanelled facility nearby*  *4- Do not trust it/government/ program*  *5- Other, specify: _____________________*  *99- Don’t know* |
| If yes in 3.1.5:  --------------------   - - - 1. At which facility/facilities did you use the Sehat Card/Sehat Sahulat Program? [multiple answers possible]   *1- Public primary care facility (rural health centers, BHU)*  *2- Private primary care facility (private clinics, doctors)*  *3- Public secondary care facility (THQ, DHQ, specialized hospitals such as maternity hospitals)*  *4- Private secondary care facility (private hospitals)*  *5- Others, please specify: _____________________________________* |
| - - 1. For what type of care/ for which purpose did you/your household use the Sehat Card/Sehat Sahulat Program? [multiple answers possible]   *1- Pregnancy/ Child Birth Related*  *2- Illness/ Disease*  *3- Accident / Injury*  *4- Other, please specify: _________________________*  *99- Don’t know* |
| - - 1. Did your household ever want to use the Sehat Card/Sehat Sahulat Program and could not?   *1- Yes* [proceed with 3.1.7.1]  *0- No* [proceed with 3.1.8]  *99- Don’t know* |
| If yes in 3.1.7: -------------------   - - - 1. What was the reason for the last time that your household could not use the Sehat Card/Sehat Sahulat Program? [don’t read options; multiple select]   *1- No staff at the help desk*  *2- Facility did not accept card/ insurance*  *3- Did not bring Sehat card with us*  *4- Treatment needed was not covered [continue to 3.1.7.1.1]*  *5- Coverage limit was too low/ used up*  *6- Card expired*  *7- Other, please specify: ______________________*  *99- Don’t know* |
| If “4” in 3.1.7.1   - - - - 1. What kind of treatment was needed, but not covered that last time?   1- inpatient (admission required)  2- outpatient (without admission)  99- don’t know |
| - - 1. On a scale from 1 (very bad) to 10 (very good), how would you rate your overall experience with the Sehat card/ Sehat Sahulat Program?  | 1  Very bad | 2 | 3 | 4 | 5 | 6 | 7 | 8 | 9 | 10  Very good | 99- Don’t know | | --- | --- | --- | --- | --- | --- | --- | --- | --- | --- | --- | |
| - - 1. If you could make a suggestion how to improve the Sehat card program, what would you like to change?   1- Cover more facilities  2- Higher coverage limit  3- Include a specific service, specify: ___________  4- Other, specify: ___________  5- No change needed  99- Don’t know |

# Health History and Costs

## General situation and risk perception

[timestamp]

[READ: Like I mentioned before, our research team is interested in your household’s health. I would like to talk about health related issues in more detail in the following. First, I would like to talk about health facilities in your area.]

| - - 1. I am interested to know how far away the next public primary care facility (rural health center, BHU) is from your home. Can you tell me how long it would take you to get to the next public primary care facility?   [Instruction: By this we mean the time the respondent need to reach the facility no matter which mode of transport s/he would use/ is usually available to him/her. Let respondent choose the unit, but enter in minutes.]  ……………… minutes |
| --- |
| - - 1. I am also interested to know how far away the next private primary care facility (GP doctors, clinics) is from your home. Can you tell me how long it would take you to get to the next private primary care facility?   [Instruction: By this we mean the time the respondent need to reach the facility no matter which mode of transport s/he would use/ is usually available to him/her. Let respondent choose the unit, but enter in minutes.]  ……………… minutes |
| - - 1. I am also interested to know how far away the next public (secondary/tertiary care) hospital is from your home. Can you tell me how long it would take you to get to the next public hospital?   [Instruction: By this we mean the time the respondent need to reach the facility no matter which mode of transport s/he would use/ is usually available to him/her. Let respondent choose the unit, but enter in minutes.]  ……………… minutes     - - - 1. Does this facility offer OPD services?   *1- Yes*  *2- No*  *99- Don’t know* |
| - - 1. I am also interested to know how far away the next private (secondary/tertiary care) hospital is from your home. Can you tell me how long it would take you to get to the next private hospital?   [Instruction: By this we mean the time the respondent need to reach the facility no matter which mode of transport s/he would use/ is usually available to him/her. Let respondent choose the unit, but enter in minutes.]  ……………… minutes   - - - 1. Does this facility offer OPD services?   *1- Yes*  *2- No*  *99- Don’t know* |

[Read: We just talked about the health facilities nearby your home. In the next step, I would like to hear your opinion on the chances that your household will be in the need of using the services offered in those (or other) facilities in the near future.]

| - - 1. On a scale from 1 (I totally agree) to 10 (totally disagree), how do you think about the following statement:   I worry about health shocks to my family.   | 1 total disagree | 2 | 3 | 4 | 5 | 6 | 7 | 8 | 9 | 10 Totally agree | 99 Don’t know | | --- | --- | --- | --- | --- | --- | --- | --- | --- | --- | --- | |
| --- | --- | --- | --- | --- | --- | --- | --- | --- | --- | --- | --- |
| - - 1. What do you think the chance that someone from your household will face a health event requiring being admitted to a hospital (in the upcoming year) is? How likely you think is that someone from your household will need admittance to hospital (in the next year)?  | 1  Very small chance | 2  Small chance | 3  Not small – not large | 4  Large chance | 5  Very large chance | 99  Don’t know | | --- | --- | --- | --- | --- | --- | |
| - - 1. What do you think the chance that someone from your household will face a health event requiring OPD services (in the upcoming coming year) is? How likely you think is that someone from your household will need OPD services (in the next year)?  | 1  Very small chance | 2  Small chance | 3  Not small – not large | 4  Large chance | 5  Very large chance | 99  Don’t know | | --- | --- | --- | --- | --- | --- | |

## Childbirth

[timestamp]

[Read: One health event where visiting a health facility is considered by some people is in the case of childbirth.]

| - - 1. Did you have any childbirth in the household during last 12 months, i.e. since [display month and year X months/weeks prior to the survey date]?   *1- Yes* [proceed to 4.2.1.1]  *0- No* [proceed to section 4.3 (individual health status)] |
| --- |
| If yes in 4.2.1  ------------------   - - - 1. Did you seek assistance of any health professional at the moment of childbirth?   *1- Yes* [proceed to 4.2.1.1.2]  *0- No* [proceed to 4.2.1.1.1]  [If no in 4.2.1.1]  ----------------------   - - - - 1. What was the main reason for not seeking any professional assistance at the moment of childbirth?   *1- Health facility too far away* *2- Could not afford costs of seeking health care*  *3- Too expensive travel costs 4- Thought the quality of health care to be low* *5- Waiting times too long  6- Consulted a family member / friend and advised not to seek health care 7- Consulted a health worker and advised to not seek health care 8- No permission to go to hospital  9- Did not want to go alone 10- Other, specify: _____________________________________*  *11- We saw no need for professional assistance.*  [If yes in 4.2.1.1]  ---------------   - - - - 1. What was the nature of health care you sought?   *1- Public primary care facility (health centers, BHU)*  *2- Private primary care facility (doctors, clinics)*  *3- Public secondary care facility (hospitals)*  *4- Private secondary care facility (hospitals)*  *5- At home: Lady health workers*  *6- At home: Midwives*  *7- Others, please specify: _________________________*  [if 1, 2, 3, 4 in 4.2.1.1.2]  ----------------  Did the mother stay overnight (IPD service)?  *1- Yes*  *0- No*  *99- Don’t know* |

## Health Care Utilization

[timestamp]

[Read: To get more information on your family’s health, I am now going to ask you some questions about the members of your core family. (similar to the way in the beginning of the interview)]

[Instruction: For the next sections about family health, invite other household members that are around/at home to join for brainstorming and help the respondent remember the incidents. (if that is possible and appropriate in the respective situation)]

[Tablet: round 1 is for the respondent, so replace [name X] with you / your]

| - - 1. In the last 12 months i.e. since [display month and year 12 months prior to the survey date], how many times has [name X] been admitted to a health facility or hospital?   [Instruction: Do not cover ODP treatment here.]  [Enter 99 for don’t know]  [numbers, 0-100] | - - 1. In the last 12 months, i.e. since [display month and year 12 months prior to the survey date], how many times did [name X] face an accident / illness where [name X] considered to get admitted / visit at a health facility or hospital, but did not seek for admission?   [Instruction: Note that this question refers to any incidence where the respondent feels like there was a need to seek admission at a hospital or where the person had been referred to hospital.]  [Enter 99 for don’t know]  [numbers, 0-100] | - - 1. In the last four weeks, i.e. since [display day of the week and date 4 weeks before the survey data], how many times did [name X] receive care from a health provider, OPD, or a pharmacy without being admitted to the health facility?   [Enter 99 for don’t know]  [numbers, 0-100] | - - 1. In the last four weeks, i.e. since [display day of the week and date 4 weeks before the survey data], how many times did [name X] face an accident / illness where he/she considered to seek care from a health provider, OPD, or a pharmacy (without being admitted to the facility) but did not go?   [Enter 99 for don’t know]  [numbers, 0-100*]* | - - 1. How many times has [name X] had any other health need/ health problem within the last 4 weeks, i.e. since [display day of the week and date 4 weeks before the survey data], for which he/she did not see the need to seek medical care?   [Enter 99 for don’t know]  [numbers, 0-100] | [if >0 & not 99 in ]  What did [name X] do instead?  1- Self-medication with modern medicine  2- Home remedies  3- Hakeem (Unani medicine practitioner)  4- Homeopathic practitioner  5- Islamic spiritual healer (pir or fakeer)  6-Pray  7- Nothing  8- Other, specify  99- Don’t know |
| --- | --- | --- | --- | --- | --- |

| - - 1. Does [name X] usually make the decision whether to visit a health facility?  1. *Yes* 2. *No* |
| --- |

## Inpatient Treatment

[timestamp]

[Tablet: The following questions should be asked for each household member who was reported to have used inpatient health care services within the past 12 months. If the respondent him/herself had an inpatient care visit, start with him/her.]

| [For every household member who was reported to have had used inpatient care within the past 12 months (i.e. for everyone who reported a number >0 in 4.3.1 and not 99), the remaining section on inpatient care is repeated in the form of another roster.]  You said that the following members of your household were admitted to a health facility or hospital in the last 12 months: [list and read all respective names]   - - 1. Is this correct?   *1- Yes* [proceed]  *0- No* [correct]   - Start roster - |  |
| --- | --- |
| ---------------   - - 1. What type of facility was [name X] admitted to for his/her most recent stay? [select one]   *1- Public primary care facility (health centers, BHU)*  *2- Private primary care facility (doctors, clinics)*  *3- Public secondary care facility (hospitals)*  *4- Private secondary care facility (hospitals)*  *5- Others, please specify: __________________________* | |
| - - 1. How many nights did [name X] stay at the facility for his/her most recent stay?   __________nights [integer numbers; >0 & < 500] | |
| - - 1. Did [name X] go to this facility because s/he was referred from another facility?   *1- Yes* [proceed to 4.4.4.1]  *0- No*  *99- Don’t know*  [if yes in 4.4.4]  --------------------   - - - 1. From which one?   *1- Public primary care facility (health centers, BHU)*  *2- Private primary care facility (doctors, clinics)*  *3- Public secondary care facility (hospitals)*  *4- Private secondary care facility (hospitals)*  *5- Others, please specify: ____________________*  *99- Don’t know* | |
| - - 1. Did [name X] get referred to a different facility after treatment at [insert answer from 4.4.2]?   *1- Yes*  *0- No*  *99- Don’t know*  [if yes in 4.4.5]  -------------------   - - - 1. To which one?   *1- Public primary care facility (health centers, BHU)*  *2- Private primary care facility (doctors, clinics)*  *3- Public secondary care facility (hospitals)*  *4- Private secondary care facility (hospitals)*  *5- Others, please specify: ___________________*  *99- Don’t know* | |
| - - 1. What was the main reason for [name X] to seek care this most recent time? Please provide a brief description of the health event. [Read options]   *1- Pregnancy/ Child Birth Related* [proceed to 4.4.6.1]  *2- Illness/ Disease* [proceed to 4.4.6.2]  *3- Accident / Injury* [proceed to 4.4.6.3]  99- *Don’t know*  [Tablet:] On second page:  What type of child birth related issue/illness/injury was it?  [Don’t read out the following options but ask for type of child birth related issue/illness/injury]  If 1 in 4.4.6  --------------------------------------------------------   - - - 1. What type of pregnancy/child birth related issue did […] face when seeking care this most recent time?   *1- Normal Delivery (in Urdu: baghairpechidgi k hamal)*  *2- C-Section (in Urdu: Operation se delivery hona)*  *3- Abortion / Miscarriage (in Urdu: Abbortion / Bacha Girana)*  *4- Delivery/Child Birth Complications (in Urdu: Hamal main pechidgiyan)*  *5- Intra Uterine Death (In Urdu: bachaydaani main bachyka mar jana)*  *6- Pre-Mature Labor Pains (in Urdu: hamal k dorandardhona)*  *7- Other, specify: ___________________________*  *99- Don’t know details*  If 2 in 4.4.6  -------------------------------------   - - - 1. What type of illness/disease did […] face when seeking care this most recent time?   *1- Heart Attack / Pain / Surgery of the heart (in Urdu: AarzaDil / Dilkadardhona)*  *2- Stroke (in Urdu: dimaghtakkhoonkanapohnchna / falijkahamla)*  *3- Hernia / Groin Pain (Hernia, aantkakhasiya main utarjana)*  *4- Dysfunctional Uterine Bleeding (DUB) / total abdominal hysterectomy (TAH) (in Urdu: sharamgah se khoonaanaaur bacha daaninikalwadena)*  *5- Abdominal Pain (in Urdu: Pait main dardhona)*  *6- Stone in gall bladder / Bladder operation (In Urdu: pitaykipathri / Pittayka operation)*  *7- Problems with Breathing, incl. astma (Saanslenay main maslahona)*  *8- Kidney Problem / urinary track problems (in urdu: gurdaykibemari)*  *9- Cancer / Tumor (In Urdu: Rasoli)*  *10- Chronic Obstructive Pulmonary Disease (Copd) (In Urdu: msalsalsaanskitakleefrehna)*  *11- Piles / Haermeroides (In Urdu: Bawaseer)*  *12- Diarrhea (in UrdU: maidayauraantkisozish)*  *13- Appendicitis (in Urdu: Appendix Pain)*  *14- Biopsy (Incision And Drainage) (In Urdu: Lab Test / Pus Test)*  *15- Typhoid (fever) or other fever (in Urdu: Typhoid (bukhar))*  *16- Hepatitis (in Urdu: Yarqaan)*  *17- Malaria (in Urdu: Malaria)*  *18- Diabetes (in Urdu: Sugar ki bemari)*  *19- High or low blood pressure (B.P.)*  *20- Tuberculosis (T.B.)*  *21- Mental illness / depression / psychological problems*  *22- Dental illness / teeth*  *23- Problems with eyes, eye infection, loss of vision*  *24- Polio*  *25- Stomach problems*  *26- Liver problems*  *27- Pneumonia / chest infection*  *28- Joint or bone pain / backbone problems / arthritis*  *29- Measles*  *30- Anemia*  *31- Blood infection*  *32- Problems with ears, ear infection, ear pain, loss of hearing*  *33- Headache / pains in limbs / unspecified pain*  *34- Skin problems*  *35- Epilepsy / fits 7 temporary paralysis*  *36- Family planning*  *38- Other, specify: _______________________* *99- Don’t know details*  If 3 in 4.4.6  --------------------------------------   - - - 1. What type of accident or injury did […] face when seeking care this most recent time?   *1- Traffic Accident (in Urdu: traffic hadsay)*  *2- Arm Fracture (in Urdu: HaddiTootna)*  *3- Leg Fracture*  *4- Other Fracture*  *5- Work Injury (In Urdu: )*  *6- Burned*  *7- Injuries from acts of violence*  *8- Other, specify: __________________________*  *99- Don’t know details* | |
| - - 1. This most recent time, did [name X] receive a medical prescription when/after being admitted to the hospital?   *1- Yes* [proceed to 4.4.7.1]  *0- No [proceed to 4.4.8]*  *99- Don’t know* | |
| [If yes in 4.4.7]:   - - - 1. When [name X] received that prescription, did your household purchase all the medicines listed on the prescription?   *1- Yes [proceed to 4.4.8]]*  *0- No [proceed to 4.4.7.1.1]*  *99- Don’t know* | |
| [If no in 4.4.7.1].   - - - - 1. What is the main reason for not purchasing all the medicines?   *1- Purchased only necessary medicines*  *2- Pharmacy was out of certain medicines / did not provide us with requested medicines.*  *3- Purchased only some because we did not have enough money*  *4- Did not purchase any medicines owing to a lack of money.*  *5- Received the medication from a friend.*  *99- Don’t know* | |
| ---------------  Headline: Expenditures   - - 1. How much money did your household spend on treatment and services that [name X] received during his/her most recent stay in the health facility/ hospital? [Instruction: do not include costs here that were reimbursed by Sehat Sahulat Program/insurance later.]     2. I am interested in all the costs for the incident, including any charges for laboratory tests, drugs, or other items. [Amount in PKR]   ____________[Tablet: enter answer with dial-pad, pre-specified at 0, no negative amounts, < 1,000,000]. | |
| -----------------------------------  Now, I would like to go through the different types of costs often associated with a stay in hospital. I would like to read out a list of items to you and I would like to know the costs attached to the respective item for [name X]’s most recent stay in the health facility/ hospital.   | ITEM | - - 1. How much did you have to pay for [ITEM] at the time of hospitalization?   AMOUNT (Rs)  [set default to 0; if don’t know enter “99”] | - - - 1. How much of the costs for [ITEM] were covered directly by the Sehat Card/ Sehat Sahulat Program/ your insurance/ any organization?   *1- All of it*  *2- Part of it*  *3- Nothing*  *4- There were no costs*  *99- Don’t know* | If 4.4.10>0   - - - 1. How much of the costs for [ITEM] were reimbursed to you later by the Sehat Card/ Sahulat Program/ your insurance/ any organization?   *1- All of it*  *2- Part of it*  *3- Nothing*  *99- Don’t know* | | --- | --- | --- | --- | | Admission form |  |  |  | | Diagnosis and treatment (medical tests and procedures) |  |  |  | | Medicines |  |  |  | | Prescriptions, reports or any other documents received from doctor |  |  |  | | Transport |  |  |  | | Costs for meals and/or accommodation incl. for bed sheets |  |  |  | | Other cost  Specify: ____________ |  |  |  | | |
| [Tablet: Calculate and display sum of all answers to 4.4.10. Display answer to 4.4.9]  [Check: answer to 4.4.9 <= sum of all 4.4.10 +20%]  [Note: the sum of OOP payments larger than the total amount the respondent reported for that hospital stay earlier. Please go back and check. ]  [if 4.4.10>0 for at least one item (i.e. hh had to pay for (parts of) treatment) &), ask the remaining questions in this block; otherwise, either move to the next hh member with an inpatient care stay or move to the next section if this was the last person with an inpatient care need] | |
| [if 3.0.3.1 = 1 (i.e. hh enrolled in Sehat Sahulat program and respondent is aware of it]   - - 1. Why did your household have to pay for the mentioned item(s) yourself/ out-of-pocket even though you have the sehat card? [multiple answers possible]   *1 – Item(s) not part of insurance coverage/ not covered by Sehat Card/Sehat Sahulat Program*  *2 – Facility not part of the empanelled hospitals*  *3 – Staff did not accept insurance/Sehat Card/ Sehalt Sahulat Program*  *4 – Insurance company/ Sehat Card/ Sehat Sahulat Program refused reimbursement*  *5 – Did not try to use insurance/Sehat Card/Sehat Sahulat Program*  *6 – Did not try to get reimbursement*  *7 – Other, please specify: ___________________________*   - - 1. How did your household finance the health expenses for [name X]’s treatment that you had to pay yourself/ out-of-pocket? [multiple select]   *1- Reducing consumption (except food)*  *2- Reducing food intake*  *3- Earning extra income through additional jobs /casual labor / extra hours worked*  *4- Using savings*  *5- Asking for donations from family, friends or neighbors*  *6- Taking a loan from a money-lender*  *7- Taking a loan from a relative / friend*  *8- Taking a loan from a bank*  *9- Postpone debt repayment*  *10- Sell or Pawn household assets used in income generation*  *11- Selling or Pawn consumption assets*  *12- Calling on specific traditional institutions*  *13- Selling key production assets, such as livestock and land*  *14- Outmigration / move to another place / flee*  *15- Taking children out of school*  *16- Other Strategy, specify: ______________________________*  *99- Don’t know*  ----- end roster----- | |

## Outpatient Treatment

[timestamp]

[Tablet: The following questions should be asked for each family member who was reported to have used OPD health care services within the past 4 weeks. The name of the household members should be available because of the household definition. If the respondent him/herself had an outpatient care visit, start with him/her.]

| [For every family member who was reported to have had used OPD care within the past 4 weeks (i.e. for everyone who reported a number>0 and not 99 in 4.3.3, ask all questions with “++++++“ ]  You said that the following members of your household received care from a health provider, OPD, or a pharmacy without being admitted to the health facility: [list and read all respective names]   - - 1. Is this correct?   *1- Yes* [proceed]  *0- No* [correct]   - Start roster - |
| --- |
| ++++++++++   - - 1. How many of [name X]’s OPD visits were related to: (Please provide a brief description of the health event)   *1- Pregnancy/ Child Birth Related: ________*  *2- Illness/ Disease: ___________*  *3- Accident / Injury: __________*  *4- Check up/ Preventive Care: ____________*  [Tablet: for each of the categories with at least one visit]  On second page:   - - - 1. What type of child birth related issue/illness/injury/check up was it? [multiple select]   [Don’t read out the following options but ask for type of child birth related issue/illness/injury/check up]  [for 1-3 same categories as for inpatient]  If 4- Check Up / Preventive Care in 4.5.2  ------------------------------------------------------  *1- Routine (preventative) check up*  *2- Check up on chronic disease*  *3- Post-treatment care visit*  *4- Vaccination / Immunization*  *5- Other, specify: __________________* |
| +++++++++++++++   - - 1. How many times did [name X] go to each of the following facility types: [table]   *1- Public primary care facility (health centers, BHU)*  *2- Private primary care facility (doctors, clinics)*  *3- Public secondary care facility (hospitals)*  *4- Private secondary care facility (hospitals)*  *5- Pharmacist*  *6- Traditional healer*  *7- Others, please specify: ____________________*  *8- Unknown facility type*  *99- Don’t know* |
| +++++++++++++++  If 4.3.3 >1   - - 1. Which of the cases was the most recent visit of [name X] to an OPD facility?   [Tablet: Display all reasons from 4.5.2 & all facilities from 4.5.3]  [Instruction: select the combination that was the most recent case for the following questions and ask all questions with ------- for this case] |
| -----------------   - - 1. During [name X]’s most recent OPD visit, did [name X] receive a medical prescription?   *1- Yes [proceed to 4.5.5.1]*  *0- No [proceed to 4.5.6]*  *99- Don’t know* |
| -----------------  [If yes in 4.5.5]:  ---------------------   - - - 1. When [name X] received that prescription, did your household purchase all the medicines listed on the prescription?   *1- Yes* [proceed to 4.5.6]  *0- No* [proceed to 4.5.5.2]  *99- Don’t know* |
| -----------------  [If no in 4.5.5.1] --------------------------   - - - 1. What is the main reason for not purchasing all the medicines?   *1- Purchased only necessary medicines*  *2- Pharmacy was out of certain medicines / did not provide us with requested medicines*  *3- Purchased only some because we did not have enough money*  *4- Did not purchase any medicines owing to a lack of money*  *5- Received the medication from a friend*  *99- Don’t know* |
| -----------------  Headline: Expenditures   - - 1. How much money did your household spend on treatment and services for [name X]’s most recent OPD visit? I am interested in all the costs including consulting fee and any expenses for other items including tests and drugs.   [Amount in PKR]  ___________[Tablet: enter answer with dial-pad, pre-specified at 0, no negative amounts, < 1,000,000).] |
| -----------------------------------   - - 1. Now, I would like to go through the different types of costs often associated with visiting a facility to receive OPD care. I would like to read out a list of items to you and I would like to know the costs attached to the respective item for [name X]’s most recent OPD visit.  | ITEM | - - - 1. How much did [ITEM] cost?   AMOUNT (Rs)  [set default to 0 and if don’t know enter “99”] | | --- | --- | | Admission form |  | | Diagnosis and treatment (medical tests and procedures) |  | | Medicines |  | | Prescriptions, reports or any other documents received from doctor |  | | Transport |  | | Other cost  Specify: ____________ |  | |  |  | |
| [Tablet: Calculate and display sum of all answers to 4.5.7. 1. Display answer to 4.5.6]  [Check: answer to sum of all 4.5.7.1 = 4.5.6. +/- 20%]  [Note: the sum of OOP payments larger than the total amount the respondent reported for OPD visit earlier. Please go back and check. ] |
| __________________________________________________________________________________  [If any 4.5.7.1>0]  ----------------------   - - - 1. Were any of these costs paid or reimbursed to you by the Sehat Card/ Sehat Sahulat Program/ any organization?   *1- Yes*  *0- No*  *99- Don’t know* |
| - - 1. How did your household finance the health expenses for [name X]’s treatment that you had to pay yourself/ out-of-pocket? [multiple select]   *1- Reducing consumption (except food)*  *2- Reducing food intake*  *3- Earning extra income through additional jobs /casual labor / extra hours worked*  *4- Using savings*  *5- Asking for donations from family, friends or neighbors*  *6- Taking a loan from a money-lender*  *7- Taking a loan from a relative / friend*  *8- Taking a loan from a bank*  *9- Postpone debt repayment*  *10- Sell or Pawn household assets used in income generation*  *11- Selling or Pawn consumption assets*  *12- Calling on specific traditional institutions*  *13- Selling key production assets, such as livestock and land*  *14- Outmigration / move to another place / flee*  *15- Taking children out of school*  *16- Other Strategy, specify: ____________________________*  *99- Don’t know* |

- End roster –

## Neglected Health Care

| [timestamp]  Headline: Neglected Health Care (Inpatient)  [Here, every household member is included who was reported to have had neglected inpatient care within the past 12 months (i.e. for everyone who war reported a number >0 and not 99 in 4.3.2 ). ]   - - 1. You said that the following members of your household faced an accident/illness where they considered being admitted to a health facility or hospital in the last 12 months but did not seek for admission: [list and read all respective names]   Is this correct?  *1- Yes* [proceed with Error: Reference source not found]  *0- No* [correct] |
| --- |
| [if yes in 4.6.1] [ask all questions with ###### for this case/household member] |
| ###########   - - 1. Thinking about the last time this happened, what was the main reason for [name X] to consider visiting a health facility / hospital but not doing so? Please describe the health condition.   *1- Pregnancy/ Child Birth Related*  *2- Illness/ Disease*  *3- Accident / Injury*  [Tablet:] On second page:   - - - 1. Please classify from the following: / What type of child birth related issue/illness/injury was it?   [Don’t read out the following options but ask for type of child birth related issue/illness/injury]  [same as for IPD care usage] |
| ###########   - - 1. What was the main reason for not seeking admission at a health facility / hospital at that time for [name X]? [don’t read options]   (Dropdown Menu:)  *1- Hospital too far away* *2- Could not afford costs of treatment in hospital*  *3- Too expensive travel costs 4- Thought the Quality of treatment in hospital to be low**5- Waiting times too long  6- Consulted a family member / friend and advised not to seek inpatient care 7- Consulted a health worker and advised to not seek inpatient care 8- No permission to go to hospital*  *9- Did not want to go alone 10- Thought would get better using traditional herbs 11- Though it would get better using medicine that we already had 12- Other, specify: _______________________________* |
| Headline: Neglected Health Care (Outpatient)  [Here, every household member is included who was reported to have had neglected outpatient care within the past 4 weeks (i.e. for everyone who reported a number >0 and not 99 in 4.3.4).]  You said that within the past 4 weeks. the following members of your household faced an accident/illness where they considered to seek care from a health provider, OPD, or a pharmacy (without being admitted to the facility) but did not go: [list and read all respective names]   - - 1. Is this correct?   *1- Yes* [proceed]  *0- No* [correct] |
| [if yes in 4.6.4.] [ask all questions with ###### for this case/household member] |
| ###########   - - 1. Thinking about the last time this happened, what was the main reason for [name X] to consider visiting a health provider, OPD, or a pharmacy (without being admitted to the facility) when he/she did not do so?   *1- Pregnancy/ Child Birth Related*  *2- Illness/ Disease*  *3- Accident / Injury*  *4- Check up/ Preventive Care*  [Tablet:] On second page:   - - - 1. What type of child birth related issue/illness/injury/check up was it?   [Don’t read out the following options but ask for type of child birth related issue/illness/injury/check up]  [like in OPD care use] |
| ###########   - - 1. What was the main reason for not seeking care at a health facility at that time? [don’t read options]   [Dropdown Menu:]  *1- Health facility too far away* *2- Could not afford costs of treatment at health facility*  *3- Too expensive travel costs 4- Thought the quality of treatment at facility to be low**5- Waiting times too long  6- Consulted a family member / friend and advised not to seek health care 7- Consulted a health worker and advised to not seek health care 8- No permission to go to health facility  9- Did not want to go alone 10- Thought would get better using traditional herbs 11- Though it would get better using medicine that we already had 12- Other, specify: _________________________________* |

| - - 1. On a scale from 1 (I strongly disagree) to 5 (I strongly agree), how do you think about the following statement: Compared to public facilities, private health facilities offer a better (quality of) service.   *1- I strongly disagree 2- I disagree*  *3- I neither agree nor disagree 4- I agree 5- I strongly agree*  *99- Don’t know* |
| --- |
| - - 1. On a scale from 1 (I strongly disagree) to 5 (I strongly agree), how do you think about the following statement: Compared to primary health care facilities, secondary or tertiary health care facilities offer a better (quality of) service.   *1- I strongly disagree 2- I disagree*  *3- I neither agree nor disagree 4- I agree 5- I strongly agree*  *99- Don’t know* |
| - - 1. On a scale from 1 (I strongly disagree) to 5 (I strongly agree), do you think about the following statement: Compared to health facilities in villages, health facilities in cities offer a better (quality of) service.   *1- I strongly disagree 2- I disagree*  *3- I neither agree nor disagree 4- I agree 5- I strongly agree*  *99- Don’t know* |
| - - 1. On a scale from 1 (I strongly disagree) to 5 (I strongly agree), how do you think about the following statement: Access to affordable health care services is a human right.   *1- I strongly disagree 2- I disagree*  *3- I neither agree nor disagree 4- I agree 5- I strongly agree*   - - 1. *99- Don’t know* |

# End of Interview Page

[timestamp]

**Phone Number:**

I would like to note down your phone number in case we need to contact you again.

What are your mobile phone numbers?

Add another Phone Number

I would also like to note down your address, so that we can easily find your house for the next interview

(Instruction: please note down the address with as much detail as possible and if needed also a description of how to find or identify the house.)

[free text]

**[INT: READ:]**

**[Respondent Name], Thank you very much for your cooperation. Your help has been very important for us. This was the first part of our survey, we will now take a short break and continue with the second part that will take around 30 more minutes.**

[Tablet] record end time

**Note down the address of the house.**

**END OF INTERVIEW**

[Instruction: (Enumerator Feedback) Please think about the level of comprehension, the level of cooperation and additional comments you want to make about the interview while the GPS location is determined.]

Locate the GPS signal of the interview

# Enumerator Feedback

[timestamp]

[Introductory Page: Enumerator Feedback]:

- 1. Comprehension level of respondent

*1- Excellent*

*2- Very Good*

*3- Good*

*4- Fair*

*5- Poor*

- 1. Cooperation level of respondent

*1- Excellent*

*2- Very Good*

*3- Good*

*4- Fair*

*5- Poor*

- 1. Did anybody help the respondent answering the questions?

*1- Yes* (Proceed to 6.3.1)

*0- No* (Proceed to 6.4)

- - 1. Who helped answering the questions?

*1- Spouse
2- Son
3- Daughter
4- Other household member
5- Friend
99- Don’t know*

- 1. Any additional comments about specific questions or data quality?
